# Supplementary material for: Fast evaluation of the adsorption energy of organic molecules on metals via graph neural networks
Source: Nat Comput Sci. 2023 May 1;3(5):433–42. doi: 10.1038/s43588-023-00437-y (PMC10766545; doi:10.1038/s43588-023-00437-y)
Supplement: Supplementary file 1 — Supplementary Sections 1–5, Figs. 1–25 and Tables 1–17. [file 43588_2023_437_MOESM1_ESM.pdf]

nature computational science

Article

https://doi.org/10.1038/s43588-023-00437-y

Fast evaluation of the adsorption energy of organic molecules on metals via graph neural networks

In the format provided by the authors and unedited

Supplementary Sections

1 FG-dataset 3
2 Adsorption Conformational Search 4
3 BM-dataset 5
4 Model Benchmarking: External Dataset Testing 6
5 Model Benchmarking: Comparison with Other Models . . 7

Supplementary Figures

1 Graph data structure representation 10
2 Data cleaning workflow for FG-Dataset 11
3 FG-dataset graph representation performance 12
4 GAME-Net architecture 13
5 Stratified data splitting 14
6 Nested cross validation performed to assess GAME-Net generalization performance 15
7 Training process visualization 16
8 GAME-Net performance: FG-dataset E_{ads} predictions with DFT gas energy 17
9 GAME-Net performance: FG-dataset E_{ads} predictions with GAME-Net gas energy 17
10 Error distribution and standard error sorted by metal . . . 18
11 GAME-Net performance: Different adsorption sites (1/3) . 19
12 GAME-Net performance: Different adsorption sites (2/3) . 20
13 GAME-Net performance: Different adsorption sites (3/3) . 21
14 GAME-Net benchmark: fcc(100) samples 22
15 GAME-Net benchmark: fcc(110) samples 23
16 BM-dataset: Biomass molecules and metal surfaces 24
17 BM-dataset: Polyurethane molecules and metal surfaces . . 25
18 BM-dataset: Plastic molecules and metals 26
19 GAME-Net benchmark: dataset from Andersen et al. . . . 27
20 GAME-Net benchmark: dataset from García-Muelas et al. . 27
21 GAME-Net benchmark: dataset from Pablo-García et al. . 28
22 GAME-Net benchmark: Performance summary with the external datasets 29
23 GAME-Net benchmark: Error box-plot comparison between PaiNN and DimeNet++ 30
24 GAME-Net benchmark: MAE comparison between PaiNN and DimeNet++ 31

25 Cysteine example study with linear scaling relationships . . 32

Supplementary Tables

1 FG-dataset: Hydrocarbons, alcohols, aldehydes, ketones and ethers. 33
2 FG-dataset: Carbonates, carboxylic acids and esters 34
3 FG-dataset: Amines and imines 35
4 FG-dataset: Amidines 36
5 FG-dataset: Thiols, thials, thioketones and thioethers . . . 37
6 FG-dataset: Amides 38
7 FG-dataset: Oximes 38
8 FG-dataset: Carbamate esters 39
9 FG-dataset: Aromatic molecules 40
10 Hyperparameter optimization summary 41
11 FG-dataset: Cross validation MAE and SEM *per* family . . 42
12 FG-dataset: Cross validation MAE and SEM errors *per* metal 42
13 GAME-Net predictions on the BM-dataset 43
14 GAME-Net benchmark on literature datasets 44
15 GAME-Net benchmark against PaiNN, DimeNet++, and GemNet-dT 44
16 Technical comparison between GAME-Net and benchmark models 45
17 Comparison of GAME-Net predictions with experimental adsorption energies 45

Supplementary Sections

1 FG-dataset

The "functional groups" (FG)-dataset created to train GAME-Net includes 207 closed-shell molecules. The set of molecules represents the most common functional groups: for each chemical family, all the existing configurations containing up to C_4 are included, except aromatics where $C_{>4}$ are considered. The chemical families included in the FG-dataset are the following:

- Alkanes, alkenes and alkynes. **Supplementary Table 1**
- Alcohols, aldehydes, ketones and ethers (1 Oxygen). **Supplementary Table 1**
- Carbonates, carboxylic acids and esters (2–3 Oxygens). **Supplementary Table 2**
- Amines and imines (1 Nitrogen). **Supplementary Table 3**
- Amidines (2 Nitrogens). **Supplementary Table 4**
- Thiols, thials, thioketones and thioethers (1 Sulfur). **Supplementary Table 5**
- Amides and oximes (1 Oxygen + 1 Nitrogen). **Supplementary Tables 6–7**

- Carbamate esters (2 Oxygens + 1 Nitrogen). **Supplementary Table 8**

- Aromatic molecules with up to two rings containing O, N and S. **Supplementary Table 9**

For each of them, the FG-dataset contains the gas-phase molecule and the molecule adsorbed on 14 metal surfaces (Ag, Au, Cd, Co, Cu, Fe, Ir, Ni, Os, Pd, Pt, Rh, Ru, Zn). The closed-packed surface was considered for all the metals. For the fcc (100) and (110) were also computed to assess structure sensitivity.

In the database, one configuration is included for each molecule/metal combination except for the aromatics with one ring, which comprise two different configurations for each metal. This leads to a total of 3315 DFT samples in the FG-dataset.

2 Adsorption Conformational Search

Even simple $C_{2\text{-}3}$ adsorbates could have approximately 100 conformations. Thus, for the initial DFT adsorption geometries we followed a simplified conformational analysis based on the heuristic rules devised in Refs. [1, 2]. These rules can be summarised as follows:

(i). The unsaturated bonds were placed close to the surface.

(ii). Heteroatoms (O, N and S) were placed close to the surface.

(iii). Carbon tails face the surface.

(iv). If the intermediate did not converge to a reasonable structure, the molecule was readjusted manually, trying up to 6 conformations that preserve the rules (i-iii).

3 BM-dataset

The "big molecules" (BM)-dataset used for testing the GNN model includes three classes of materials:

- Biomass. Ref. [3], **Figure 16**
- Polyurethane precursors. Ref. [4], **Figure 17**
- Plastics. Ref. [5] **Figure 18**

These three groups consist of complex chemical structures that can be seen as combinations of the functional groups present in the FG-dataset. For each group, 5 representative molecules of larger size compared to those in the FG-dataset have been selected and relaxed through DFT to simulate the gas-phase and adsorption configuration on 2 metals chosen according to the existing applications and studies. The BM-dataset (45 samples, 30 adsorptions + 15 gas-phase) is used as an additional test for assessing the GNN performance on samples coming from a distribution distinct from that used to build the model (FG-dataset). For example, for the plastic group we represent polyethylene (PE), polypropylene (PP, both syndio- and iso-tactic), polystyrene (PS) and polyethylene tereftalate (PET) as molecules composed by a reasonable number of monomers. We generated the DFT adsorption systems of these molecules on Pt and Ru metal surfaces as these

represent potential candidates for applications related to chemical recycling technology.

4 Model Benchmarking: External Dataset Testing

We assessed GAME-Net performance with three literature datasets. These datasets differ significantly from the FG-dataset, as they include small open-shell fragments. The first dataset comes from Andersen et al. [6] and contains C, H, O, CH, CO, and OH adsorbed on 9 transition metals. As expected, GAME-Net performance for this dataset is poor (MAE=1.49 eV, **Figure 19**), as additionally the DFT calculations have been done with a different functional and software (Quantum Espresso). The second dataset comes from García-Muelas et al. [7] and considers C_{1-2} species adsorbed on the same metal surfaces considered in the FG-dataset (all except Fe and Co). As this dataset has been generated with the same PBE functional and software (VASP), the performance of GAME-Net improves, providing a MAE of 0.85 eV, still relatively poor as also here small open-shell fragments are considered (**Figure 20**). The third benchmark dataset is from Pablo-García et al. [8] and comprises all the intermediates formed during the CO_2 electro-reduction on copper towards C_3 products. Since the adsorbates included here are larger and present a behaviour closer to those of the closed-shell molecules of the FG-dataset, the GNN performance improves, with a MAE of 0.58 eV (**Figure 21**). Additionally, to test the performance with closed-shell molecules on different surface orientations,

we generated two additional datasets, including the same 207 molecules of the FG-dataset adsorbed on the (110) and (100) facets of the fcc metals (Ag, Au, Cu, Ir, Ni, Pd, Pt, and Rh). The model performance for the total energy reveals a MAE of 0.42 eV and 0.34 eV, respectively.

5 Model Benchmarking: Comparison with Other Models

As final test, we benchmarked GAME-Net against three GNN models, PaiNN, DimeNet++ and GemNet-dT.[9–11] The decision of using these models has been made based on their public accessibility, employing the optimized hyperparameters for the OC20 dataset.[12] Concerning the benchmark GAME-Net vs PaiNN and DimeNet++, we trained the last models by using the FG-dataset and compared their prediction power using the nested cross validation method. The performance of these models has been additionally tested against the E_{ads} of the BM-dataset. The main issues appeared during this part of the work stem from the different setups used in these models, compared to ours. First, these models consider all the slab atoms to generate the graphs, while ours takes into account only the surface ensemble formed by the adsorbate and the nearest metal atoms. The second complexity comes from the general purpose architecture of these model, since they aim at predicting the adsorption energy of the relaxed structure starting from the graph of the initial adsorption geometry, aligning themselves to the IS2RE (Initial Structure to Relaxed Energy) task of the Open Catalyst Project (OCP). In contrast to this, our GNN has

been trained with the graphs of the relaxed geometries of the adsorption systems, a fact that imposes limitations (i.e., how do we know the final graph of the relaxed system?) but also important advantages (more natural mapping between final structure and related energy, no human bias due to initial adsorbate placing, ability to test different adsorption positions). In order to perform the comparison between models as logically as possible, we considered all possible combinations to compare our work to the others in literature.

Thus, the performance of PaiNN and DimeNet++ models has been evaluated using the relaxed and unrelaxed geometries of the FG-Dataset, considering two representations for each geometry: (i) the full slab including all the surface atoms present at the surface and (ii) a reduced, ensemble structure equivalent to our graph model, as depicted in **Figure 2**, but still containing the atomic positions. The possible structural combinations sum up for total of 8 benchmarks *per* model, each being assessed following the nested cross validation methodology described in **Methods Section 2.6**. The training hyperparameters and scaling factors used to train the OC20 models have been used, considering 200 epochs for each training.[13] The MAE of the cross validation has been computed and used as the criterion to select the best model for each benchmark. The selected models have been finally used to estimate the adsorption energies, E_{ads}, of the BM-dataset by following the procedure described in **Application to industrially relevant problems** subsection of the manuscript. To preserve the coherence of the tested models, the slab energy has not been subtracted for the full slab representations, and thus it needs to be predicted by the

models to compute the E_{ads} of the BM-Dataset. An additional benchmark using GemNet-dT (an improved version of DimeNet++) using the OC22 training hyperparameters and our own scaling factor has been conducted for the unrelaxed full slab structures. The results of these benchmarks are summarized in **Supplementary Table 15** and depicted in **Figure 23-24**, while their technical differences are found in **Supplementary Table 16**.

Figures

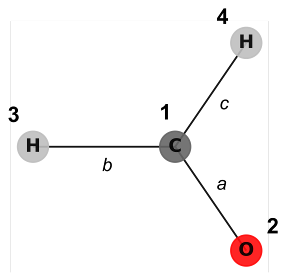

Node embedding:
One-hot encoding

**Node**	C	H	O	N	S
**1**	1	0	0	0	0
**2**	0	0	1	0	0
**3**	0	1	0	0	0
**4**	0	1	0	0	0

Graph connectivity:
COO format

**Edge**	node 1	node 2
a	**1**	**2**
b	**1**	**3**
c	**1**	**4**

Supplementary Figure 1: Example of graph data structure representation. Atoms are represented as nodes embedding the chemical element via one-hot encoding, while the connectivity is defined by the edge list.

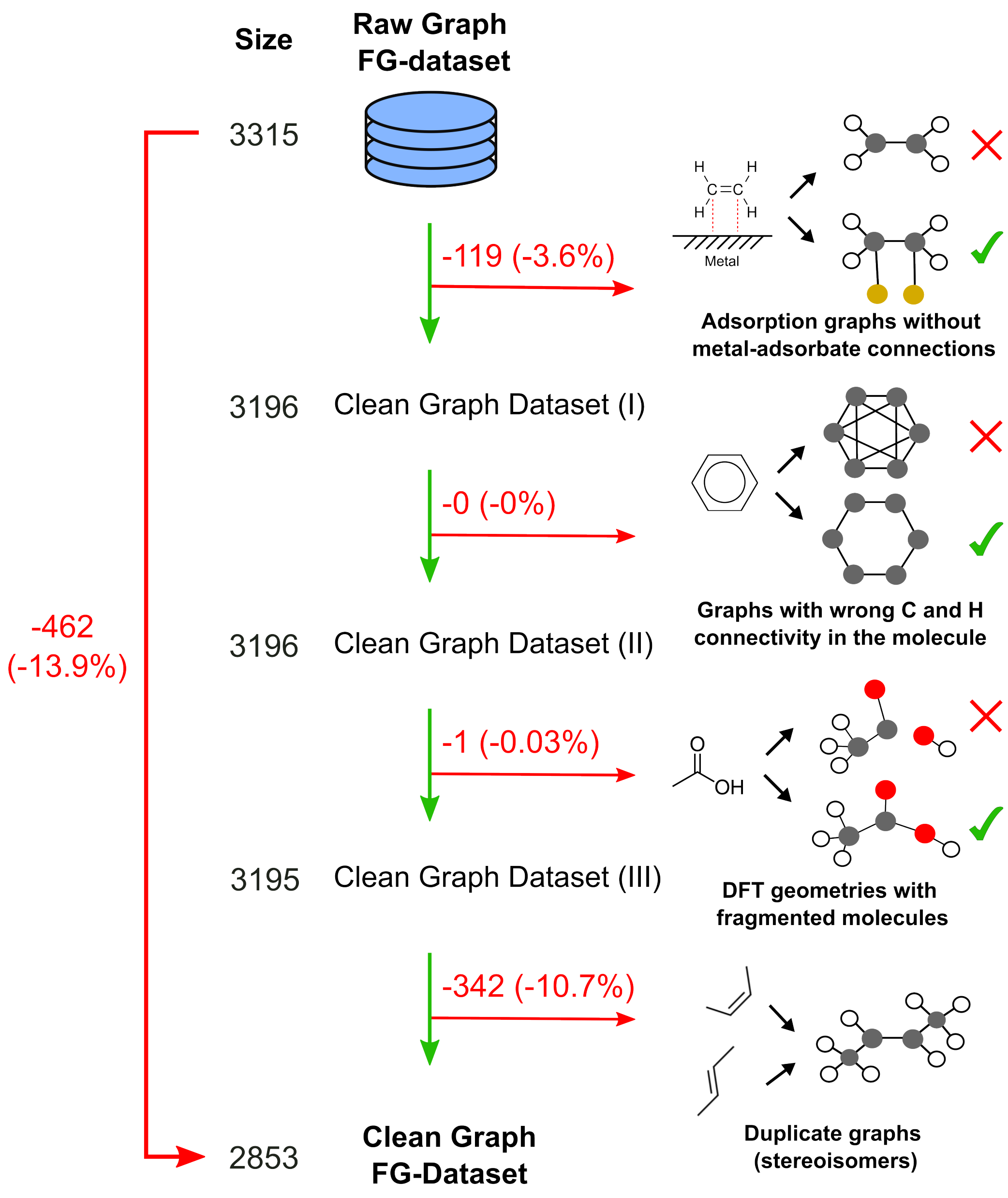

Supplementary Figure 2: Data cleaning workflow applied to the raw graph FG-dataset. The red numbers represent the number of graphs (with relative percentage compared to the previous step in parentheses) that are filtered out at each cleaning step.

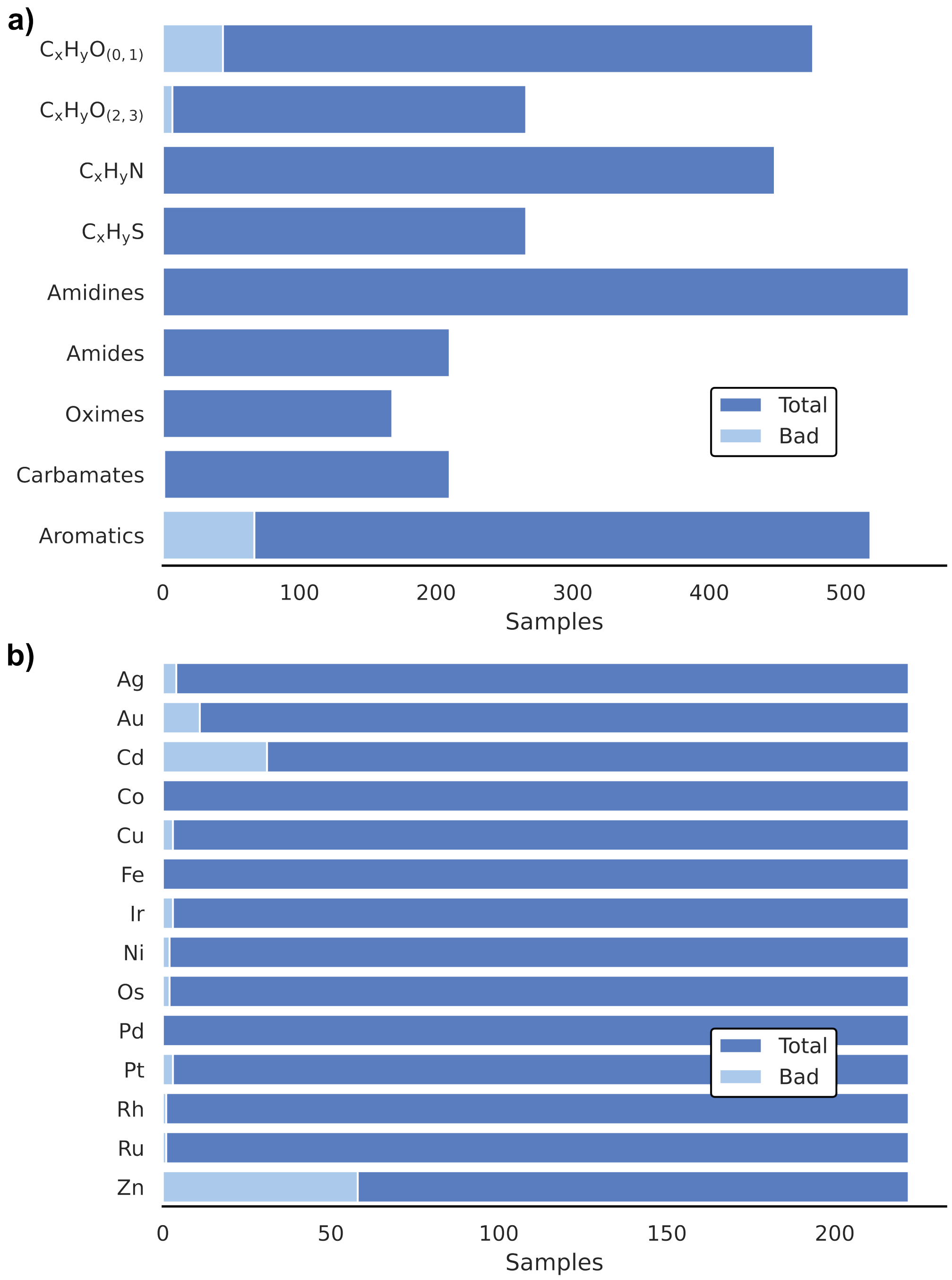

Supplementary Figure 3: Graph representations of the FG-dataset without metal-adsorbate connections. a) Distribution by functional group and b) by metal.

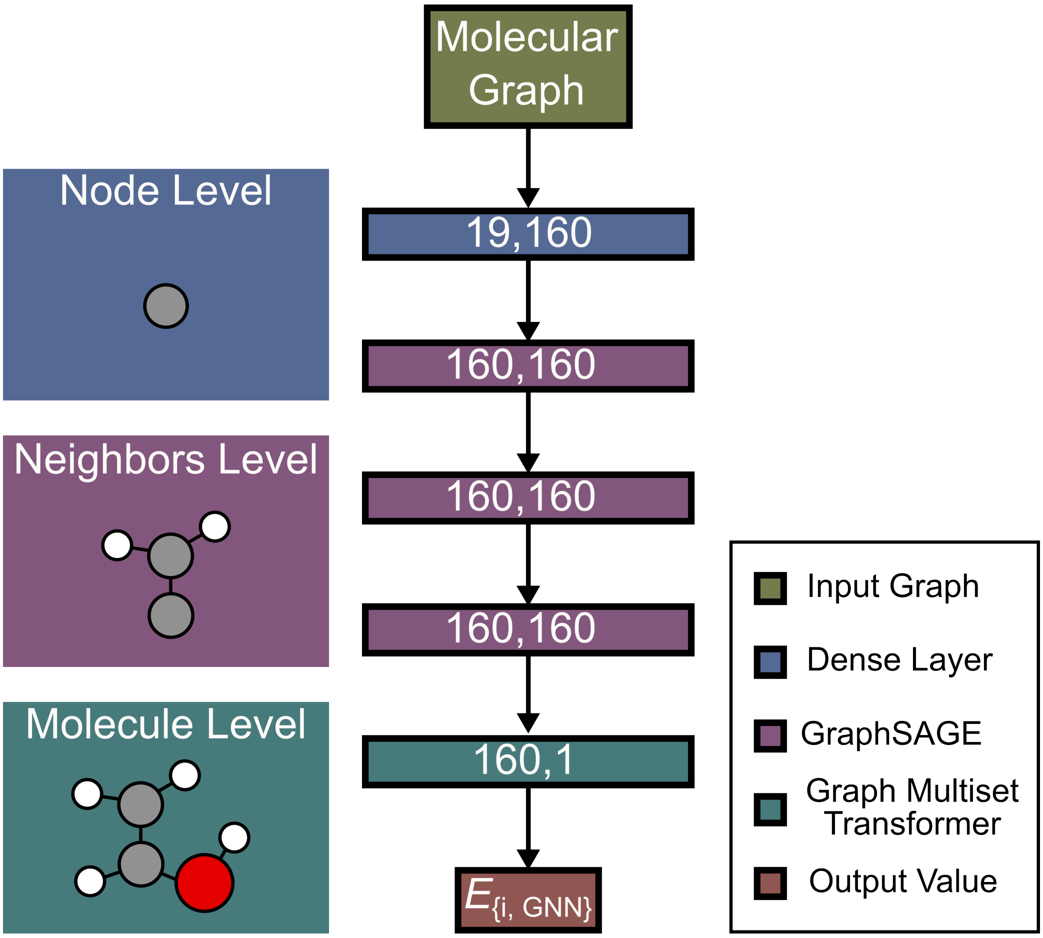

Supplementary Figure 4: GAME-Net architecture. Each block contains the initial and final dimension of the related transformation module. Note that the first input dimension matches the number of chemical elements present in the FG-dataset (14 metals + C, H, O, N, and S), and the model output is a scalar value representing the target energy.

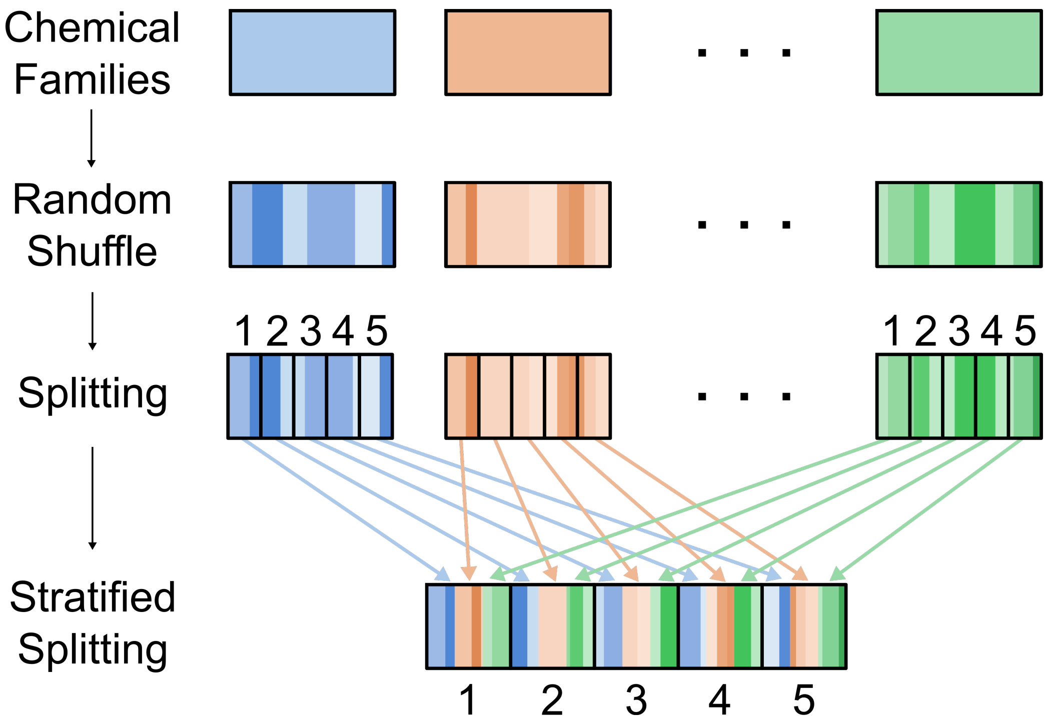

Supplementary Figure 5: Stratified data splitting. To ensure that each chemical family is equally represented among the train/val/test sets during the model training, each family subset is shuffled, split in 5 indexed blocks which are then concatenated by index to generate the final splits.

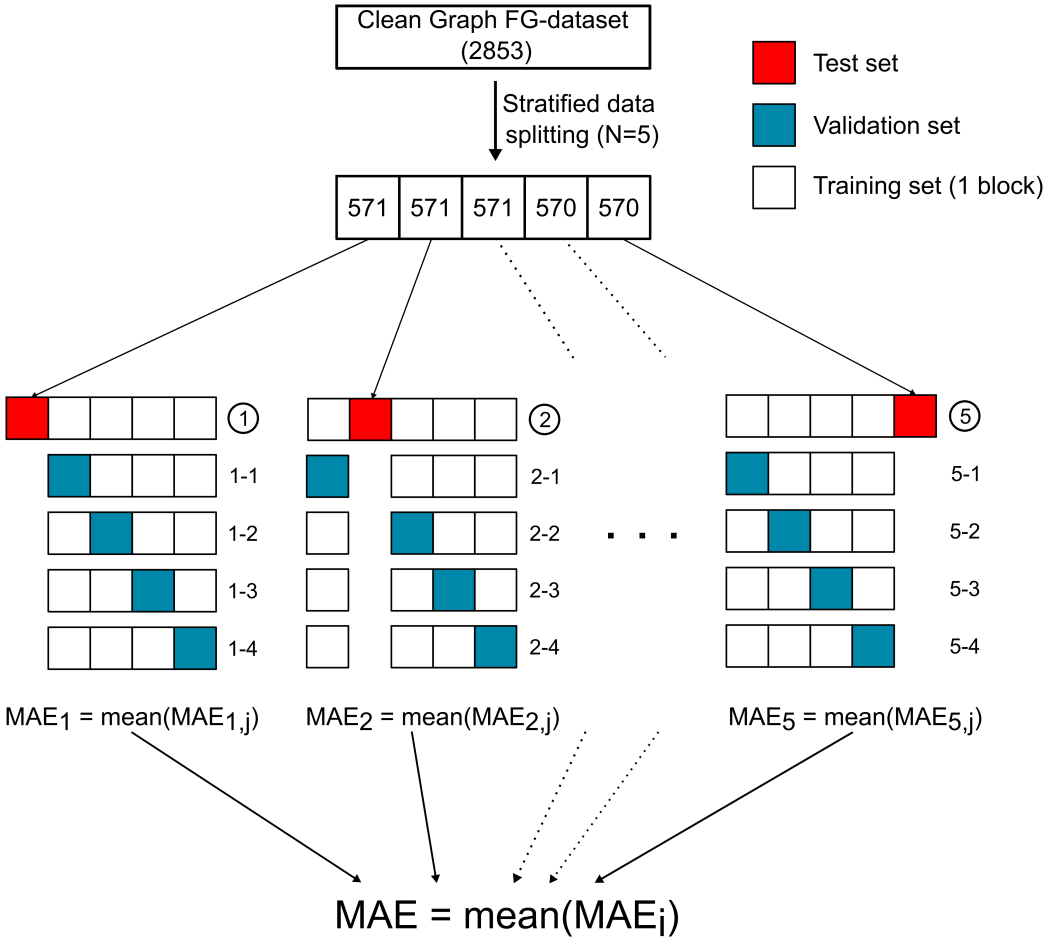

Supplementary Figure 6: Nested cross validation performed to assess GAME-Net generalization performance. The FG-dataset is split into 5 subsets and GAME-Net is trained with all the possible combinations for generating the train/val/test sets, where one subset is used as test set, a second one as validation set and the remaining three compose the training set. This procedure leads to a total of 20 training processes with unique data combinations. The final MAE is obtained by averaging all the MAEs.

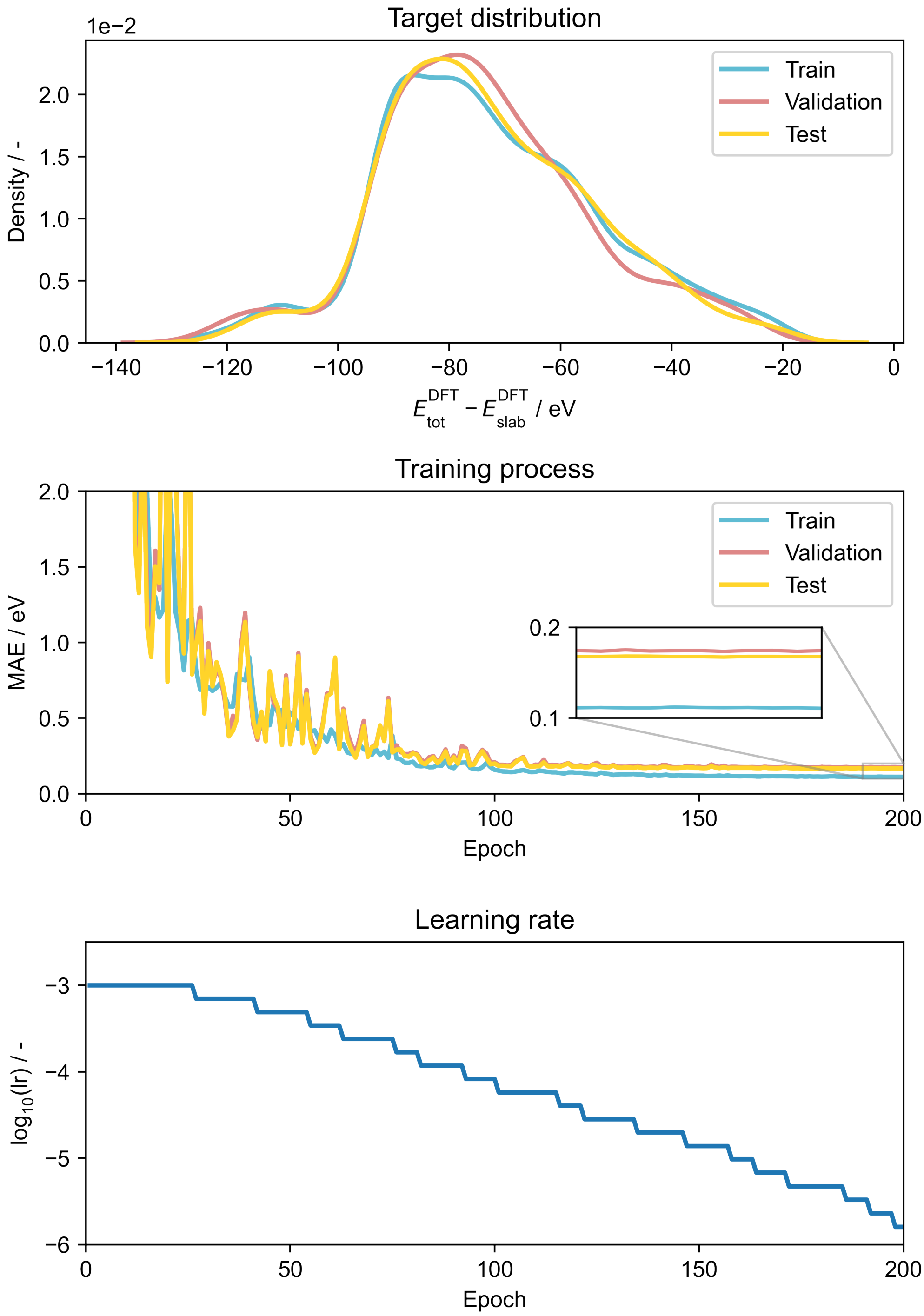

Supplementary Figure 7: Training process visualization. From top to bottom: Graph target distribution among the generated train/val/test sets, MAE trend during the training process, and related learning rate adjustment.

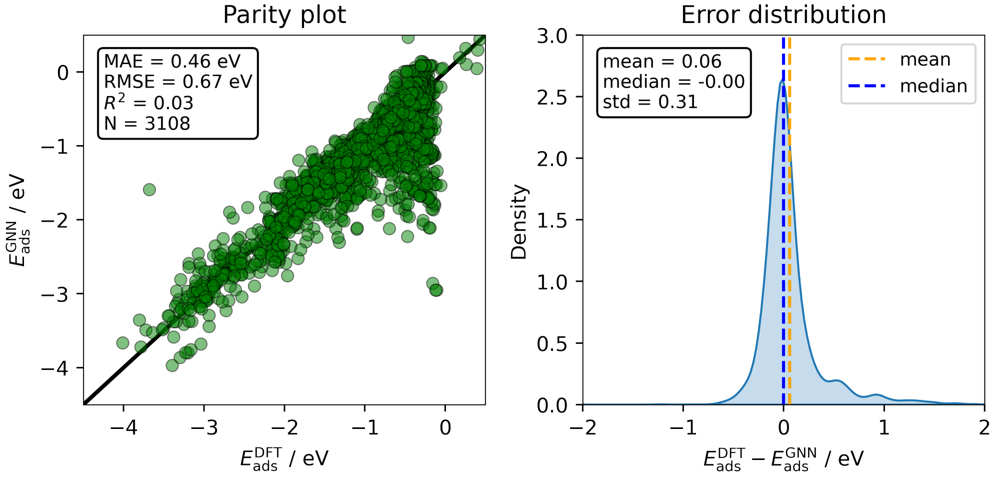

Supplementary Figure 8: GAME-Net performance: FG-dataset E_{ads} predictions with DFT gas energy. Parity plot (left) and error distribution (right) of the adsorption energy prediction of GAME-Net for the entire FG-dataset, computed subtracting the DFT gas-phase energy of the molecule.

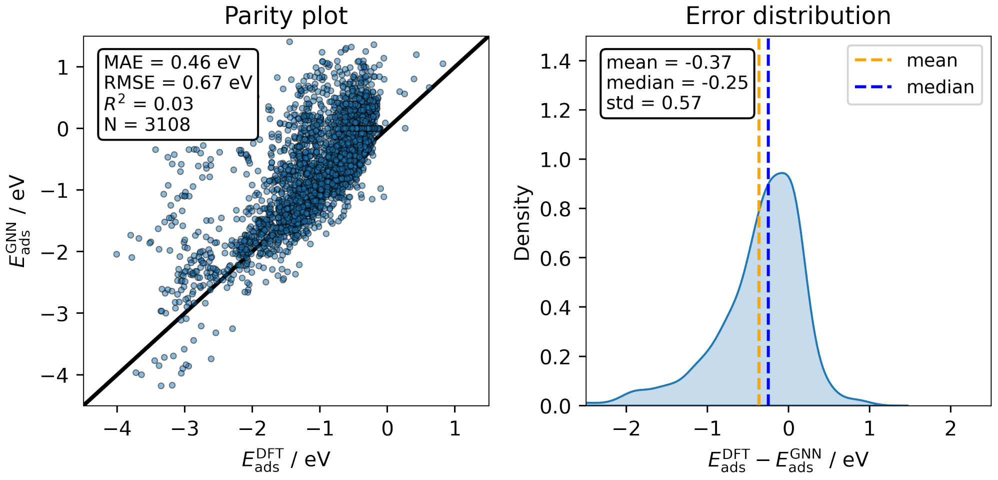

Supplementary Figure 9: GAME-Net performance: FG-dataset E_{ads} predictions with GAME-Net gas energy. Parity plot (left) and error distribution (right) of the adsorption energy prediction of GAME-Net for the entire FG-dataset, computed subtracting the GAME-Net prediction for the gas energy.

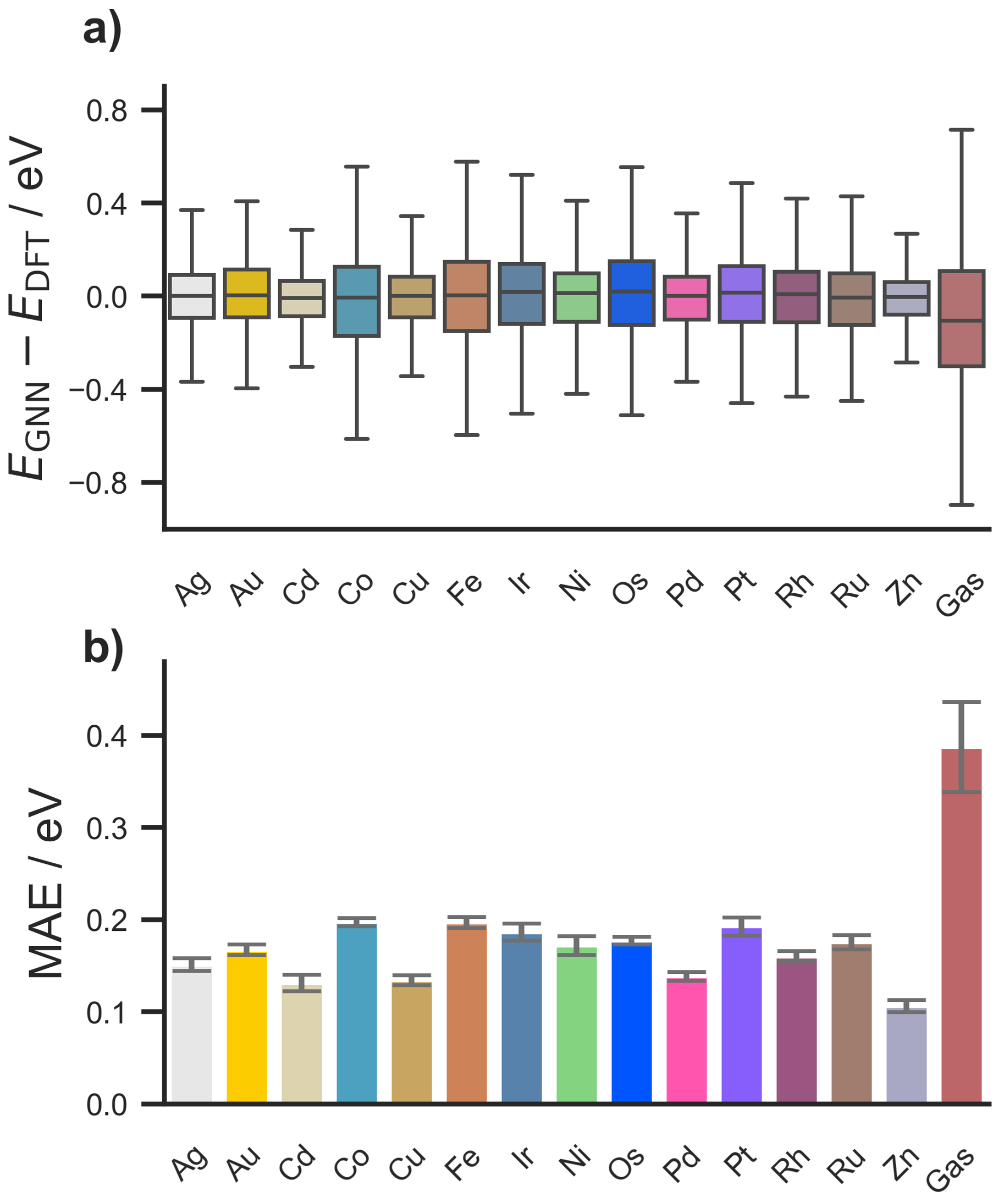

Supplementary Figure 10: Error distribution and standard error sorted by metal. a) Box-plot of the error and b) mean absolute error grouped by metal in the test sets from the 5-fold nested cross validation. The dataset size is n=11412 as the k-fold nested cross validation involves including all the FG-dataset graphs (n=2853) in the test set $k-1$ times, each one with a unique combination of train/validation sets. Box-plots in panel a define the median as box center, the interquartile range (IQR) as the box size, with whiskers extending for 1.5IQR. Data in panel b are presented as mean ± standard error.

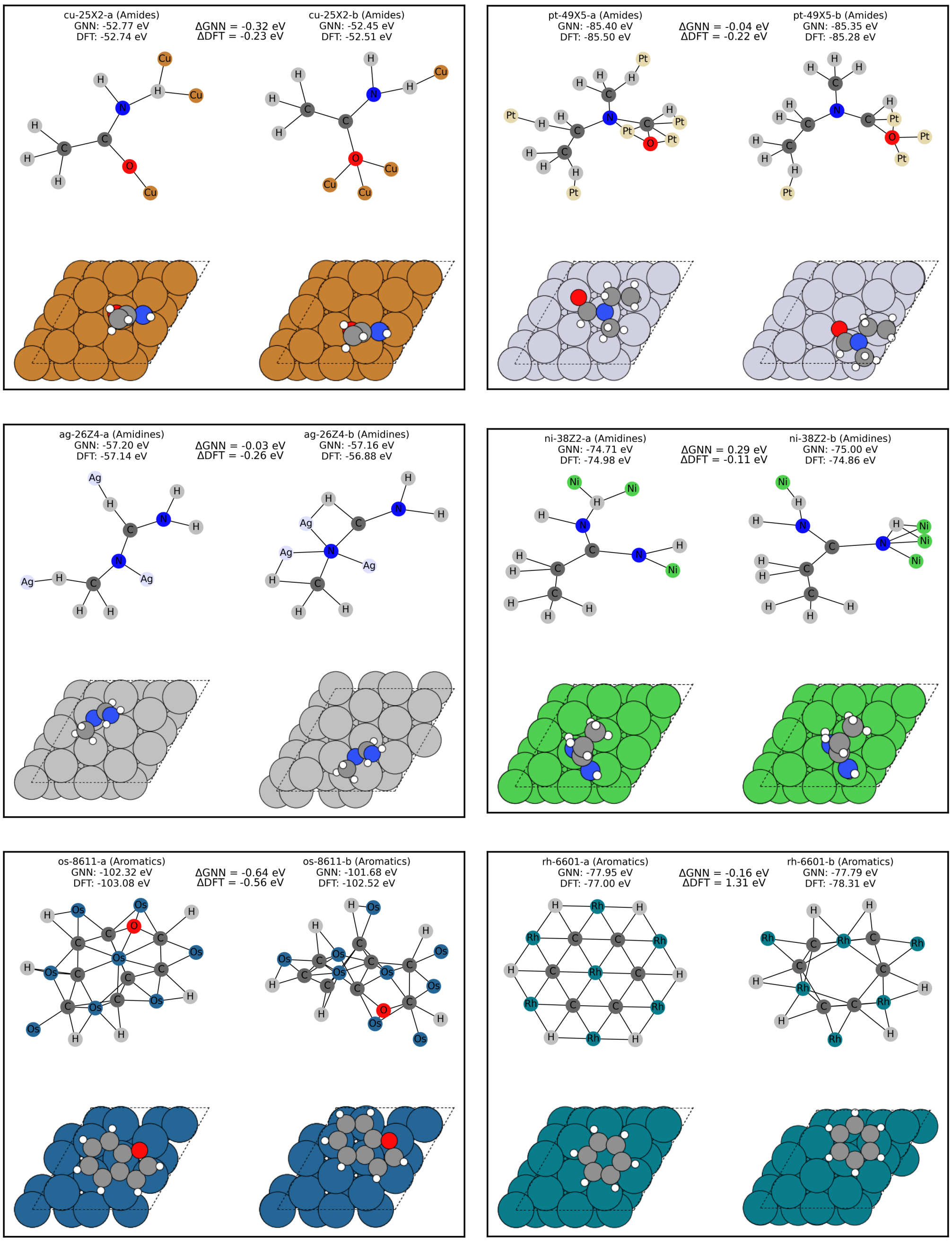

Supplementary Figure 11: GAME-Net performance: Different adsorption sites (1/3). Each panel contains two different adsorption configurations for the same adsorbate-metal pair, with the related graph representation and model prediction compared to the DFT target.

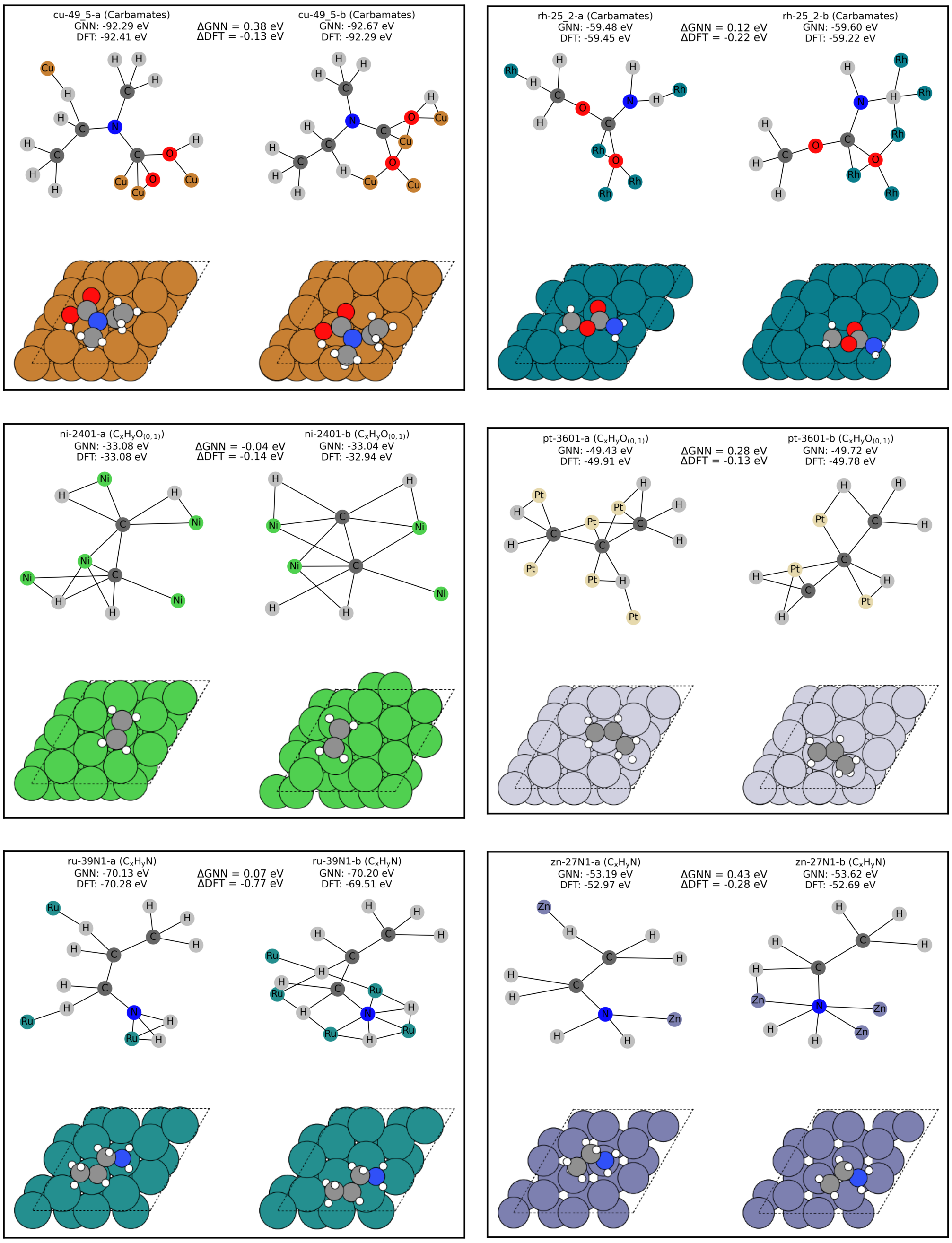

Supplementary Figure 12: GAME-Net performance: Different adsorption sites (2/3). Each panel contains two different adsorption configurations for the same adsorbate-metal pair, with the related graph representation and model prediction compared to the DFT target.

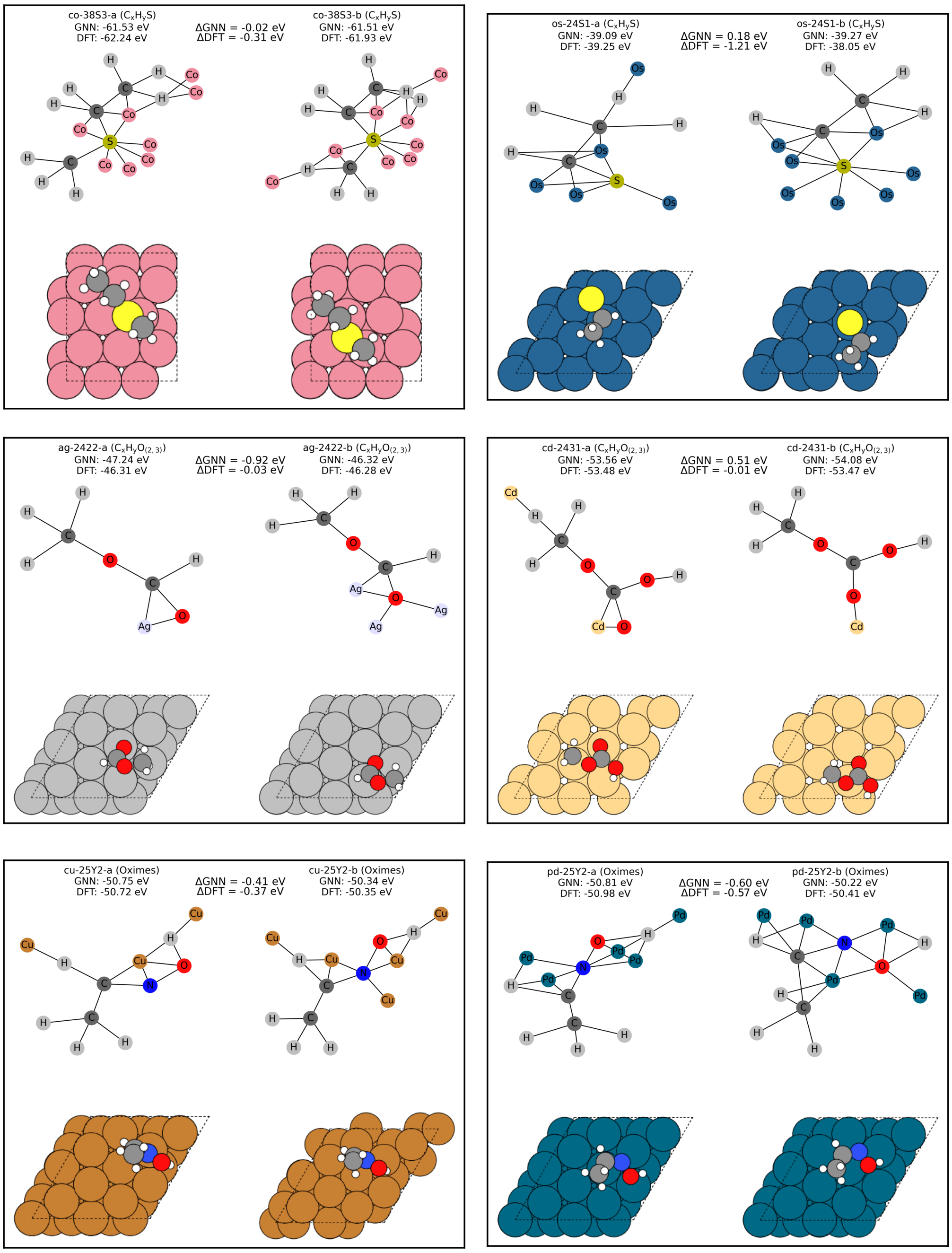

Supplementary Figure 13: GAME-Net performance: Different adsorption sites (3/3). Each panel contains two different adsorption configurations for the same adsorbate-metal pair, with the related graph representation and model prediction compared to the DFT target.

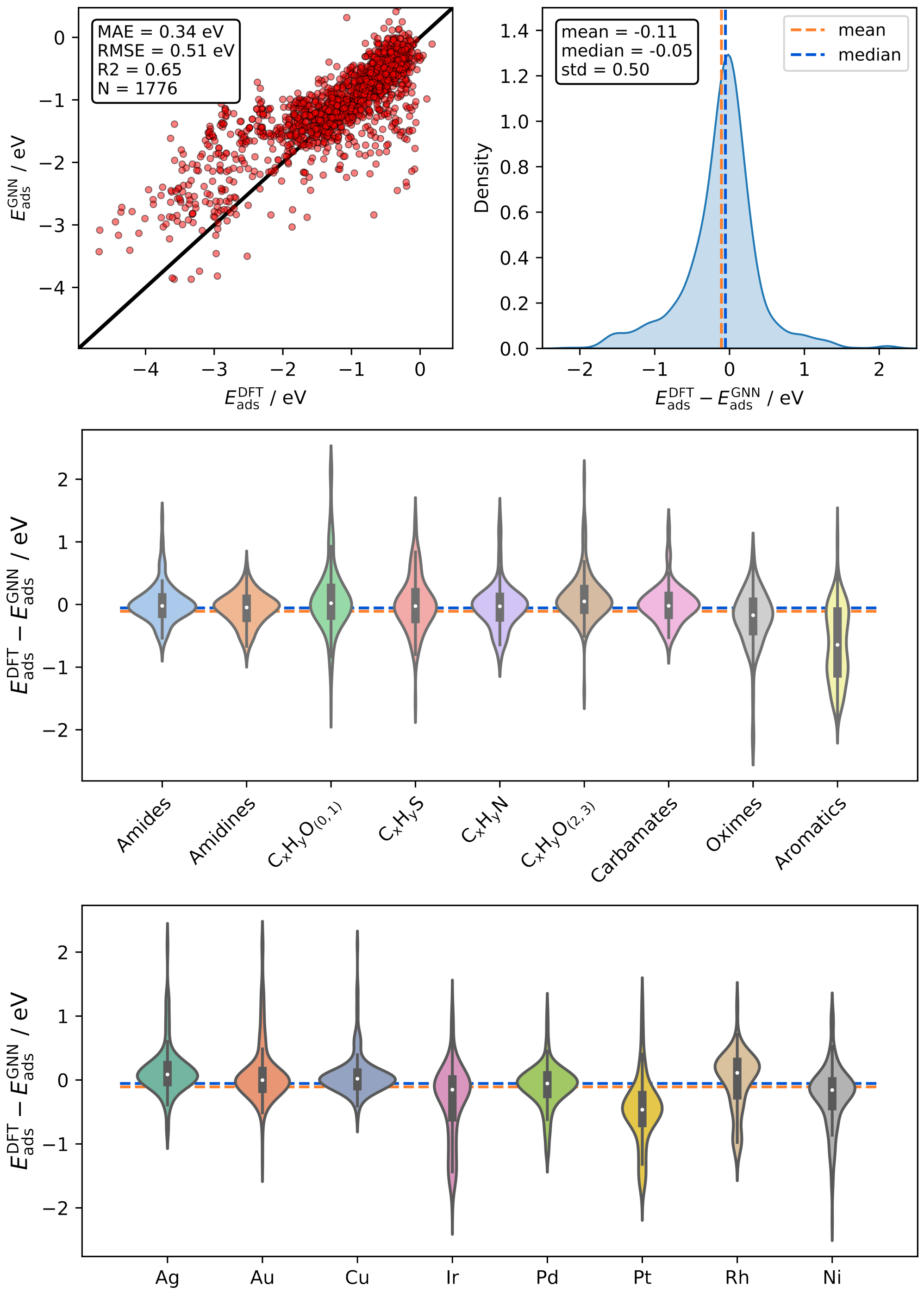

Supplementary Figure 14: GAME-Net benchmark: fcc(100) samples. From top to bottom: Parity plot (left) and error distribution (right) of the adsorption energy prediction for the FG-dataset molecules adsorbed on the fcc(100) facets, violin-plots of the error grouped by family and metal.

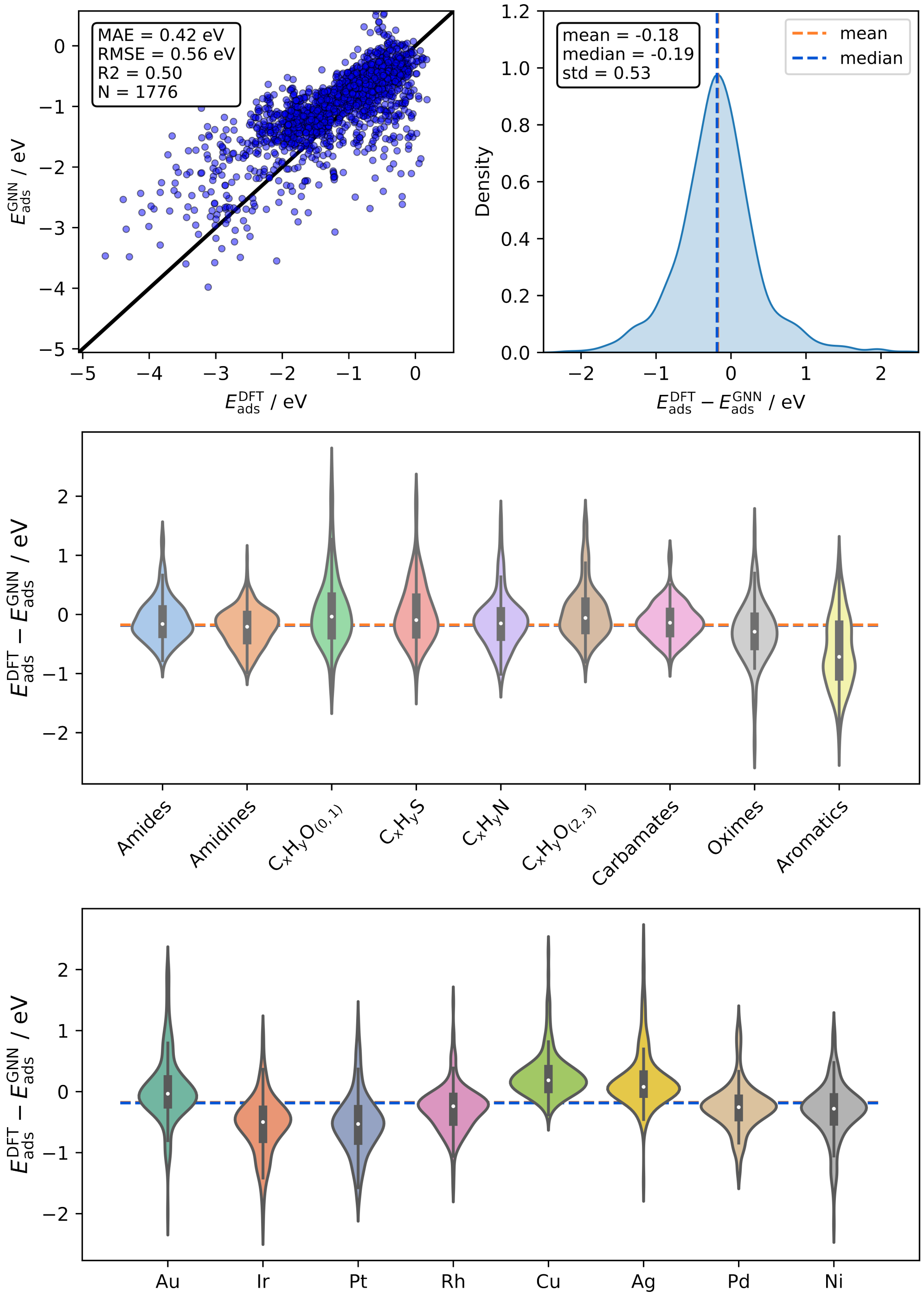

Supplementary Figure 15: GAME-Net benchmark: fcc(110) samples. From top to bottom: Parity plot (left) and error distribution (right) of the adsorption energy prediction for the FG-dataset molecules adsorbed on the fcc(110) facets, violin-plots of the error grouped by family and metal.

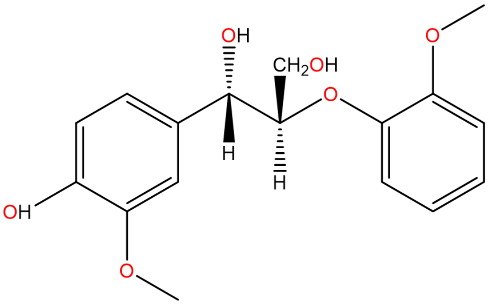

(1*S*,2*S*)-1-(4-hydroxy-3-methoxyphenyl)-2-(2-methoxyphenoxy)propane-1,3-diol

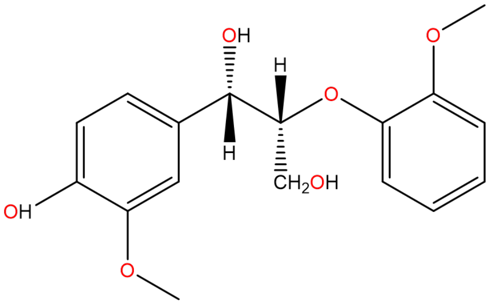

(1*S*,2*R*)-1-(4-hydroxy-3-methoxyphenyl)-2-(2-methoxyphenoxy)propane-1,3-diol

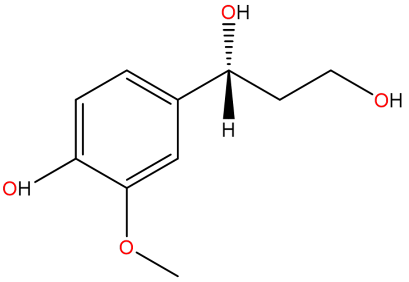

(*R*)-1-(4-hydroxy-3-methoxyphenyl)propane-1,3-diol

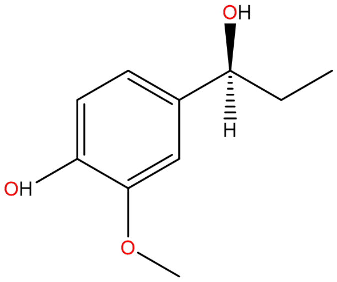

(*S*)-4-(1-hydroxypropyl)-2-methoxyphenol

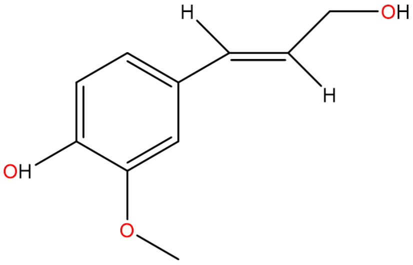

(*E*)-4-(3-hydroxyprop-1-en-1-yl)-2-methoxyphenol

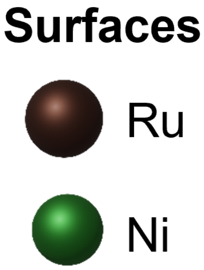

Supplementary Figure 16: BM-dataset: Biomass molecules and metal surfaces.

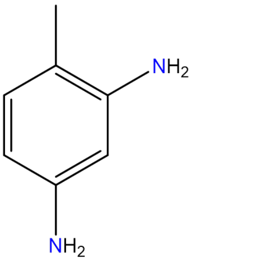

4-methylbenzene-1,3-diamine

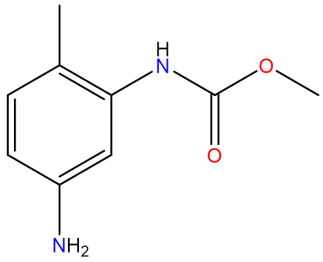

methyl (5-amino-2-methylphenyl)carbamate

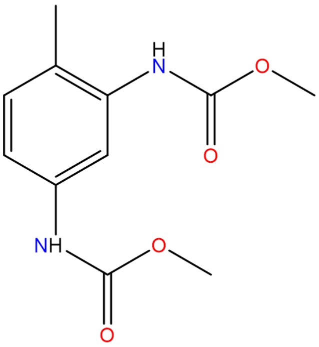

dimethyl (4-methyl-1,3-phenylene)dicarbamate

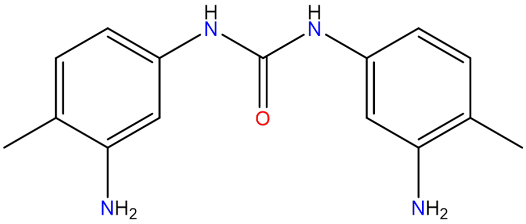

1,3-bis(3-amino-4-methylphenyl)urea

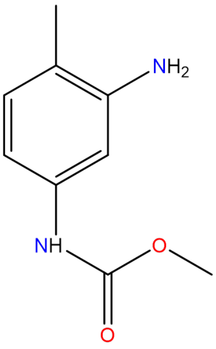

methyl (3-amino-4-methylphenyl)carbamate

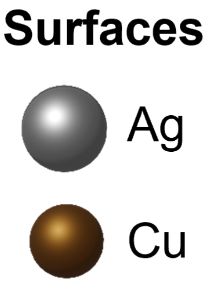

Supplementary Figure 17: BM-dataset: Polyurethane molecules and metal surfaces.

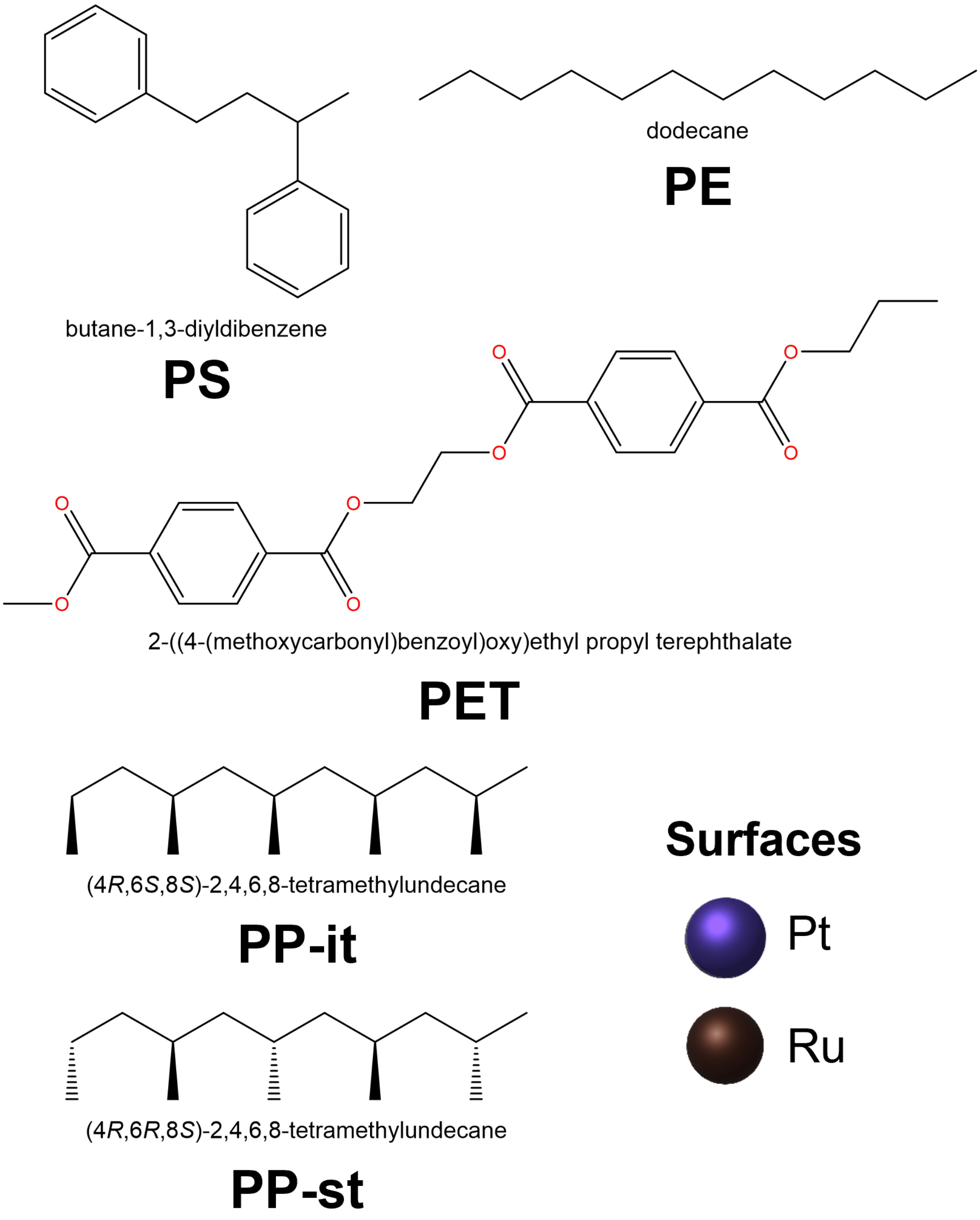

Supplementary Figure 18: BM-dataset: Plastic molecules and metals. Each molecule included in this subset represents a finite number of monomer units of the polymer depicted in bold uppercase below the respective IUPAC name.

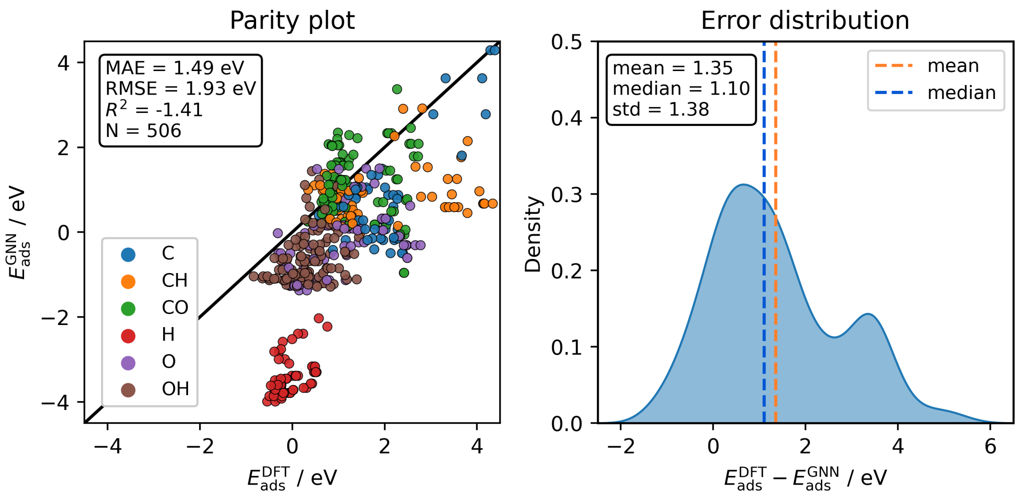

Supplementary Figure 19: GAME-Net benchmark: dataset from Andersen et al. containing open shell molecules.[6] Parity plot (left) and error distribution (right) of the adsorption energy prediction for this test dataset are depicted.

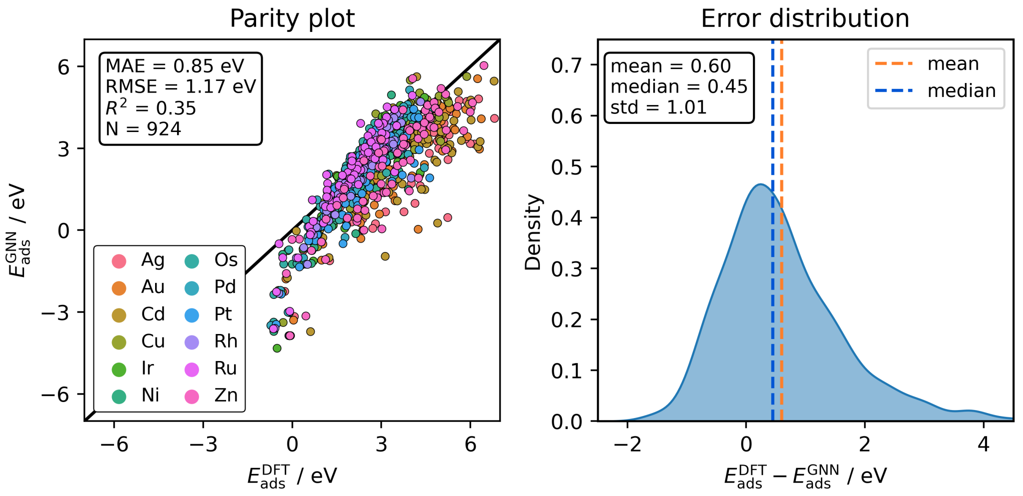

Supplementary Figure 20: GAME-Net benchmark: dataset from García-Muelas et al. containing open shell molecules.[7] Parity plot (left) and error distribution (right) of the adsorption energy prediction for this test dataset are depicted.

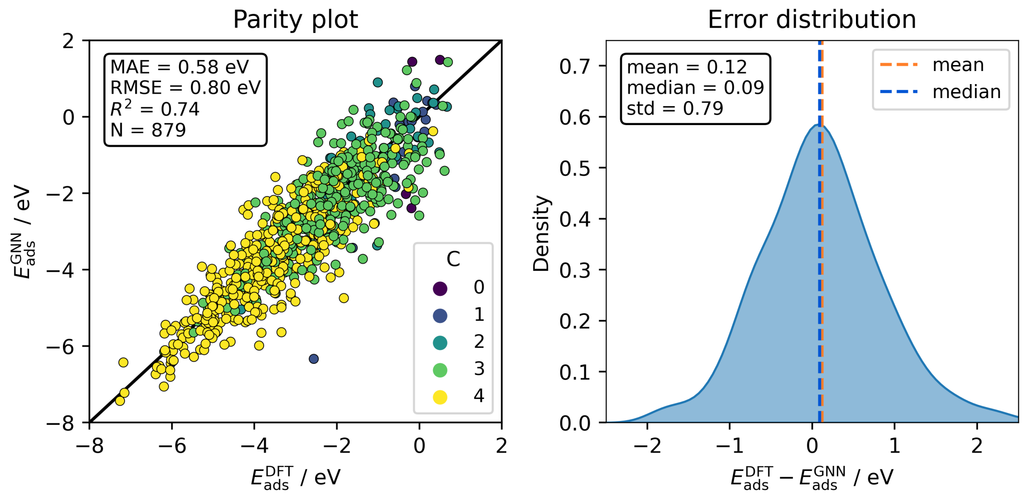

Supplementary Figure 21: GAME-Net benchmark: dataset from Pablo-García et al. containing open shell molecules.[8] Parity plot (left) and error distribution (right) of the adsorption energy prediction for this test dataset are depicted.

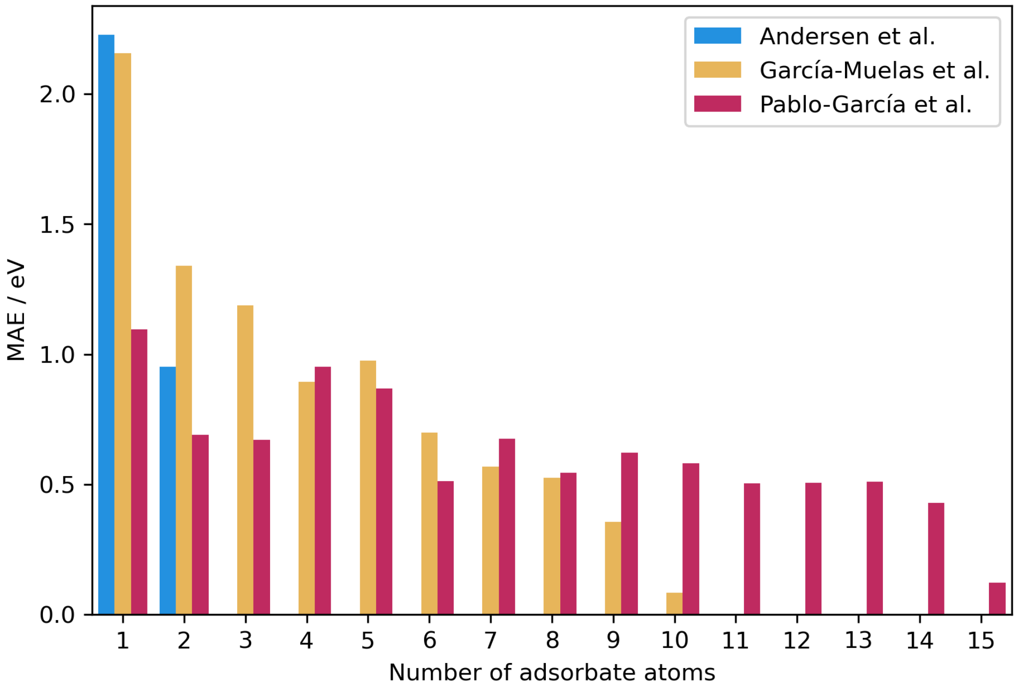

Supplementary Figure 22: GAME-Net benchmark: Performance summary with the external datasets. Mean absolute error distribution grouped by adsorbate atoms count and dataset.

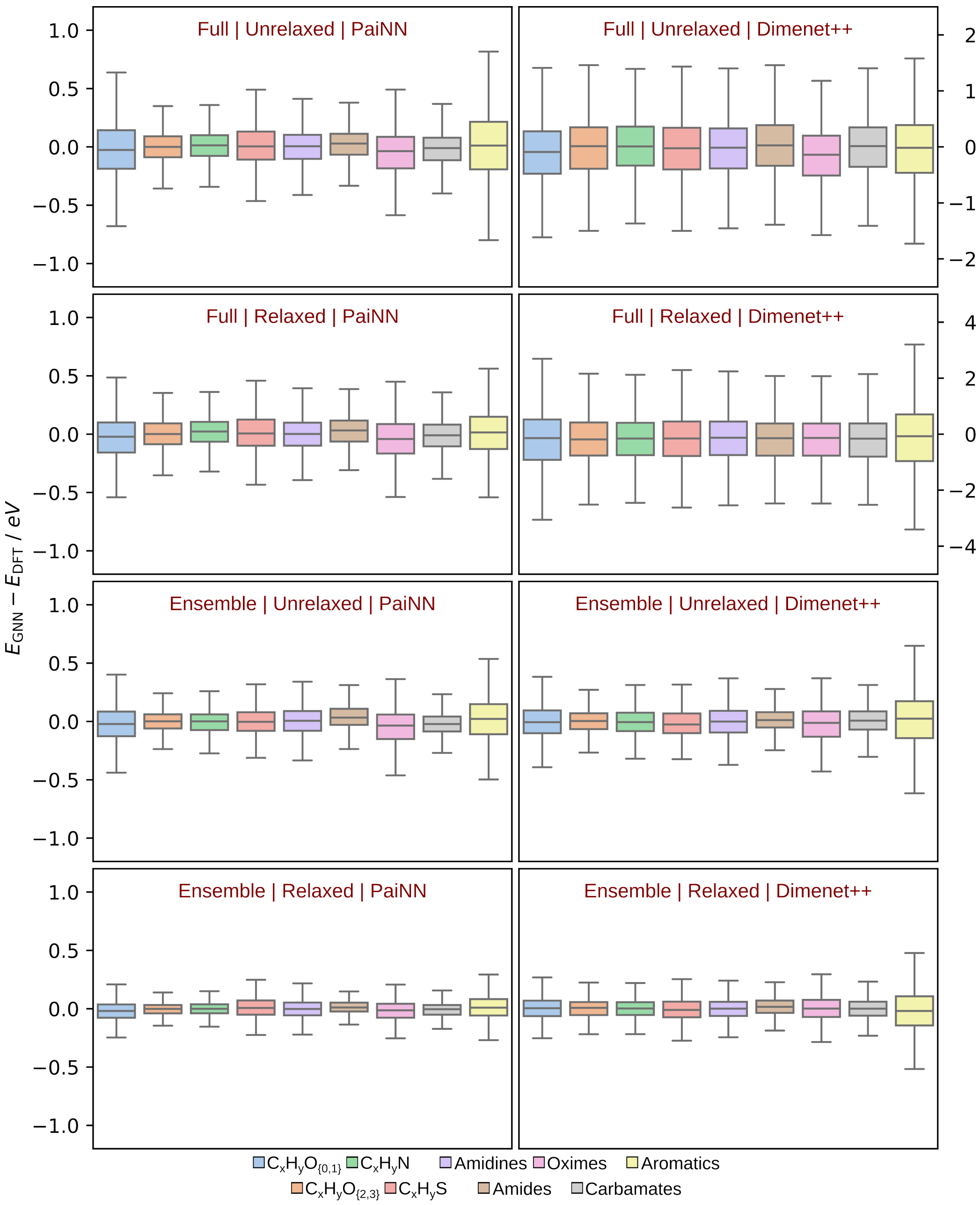

Supplementary Figure 23: GAME-Net benchmark: Comparison with PaiNN and DimeNet++. Error box-plots sorted by chemical family in the test sets from the 5-fold nested cross validation with PaiNN (left column) and DimeNet++ (right column). Each row represents a setting with specific geometry (unrelaxed/relaxed) and graph representation (full slab/ensemble). Box-plots define the median as box center, the interquartile range (IQR) as box size, with whiskers extending for 1.5IQR. The dataset size is n=13028.

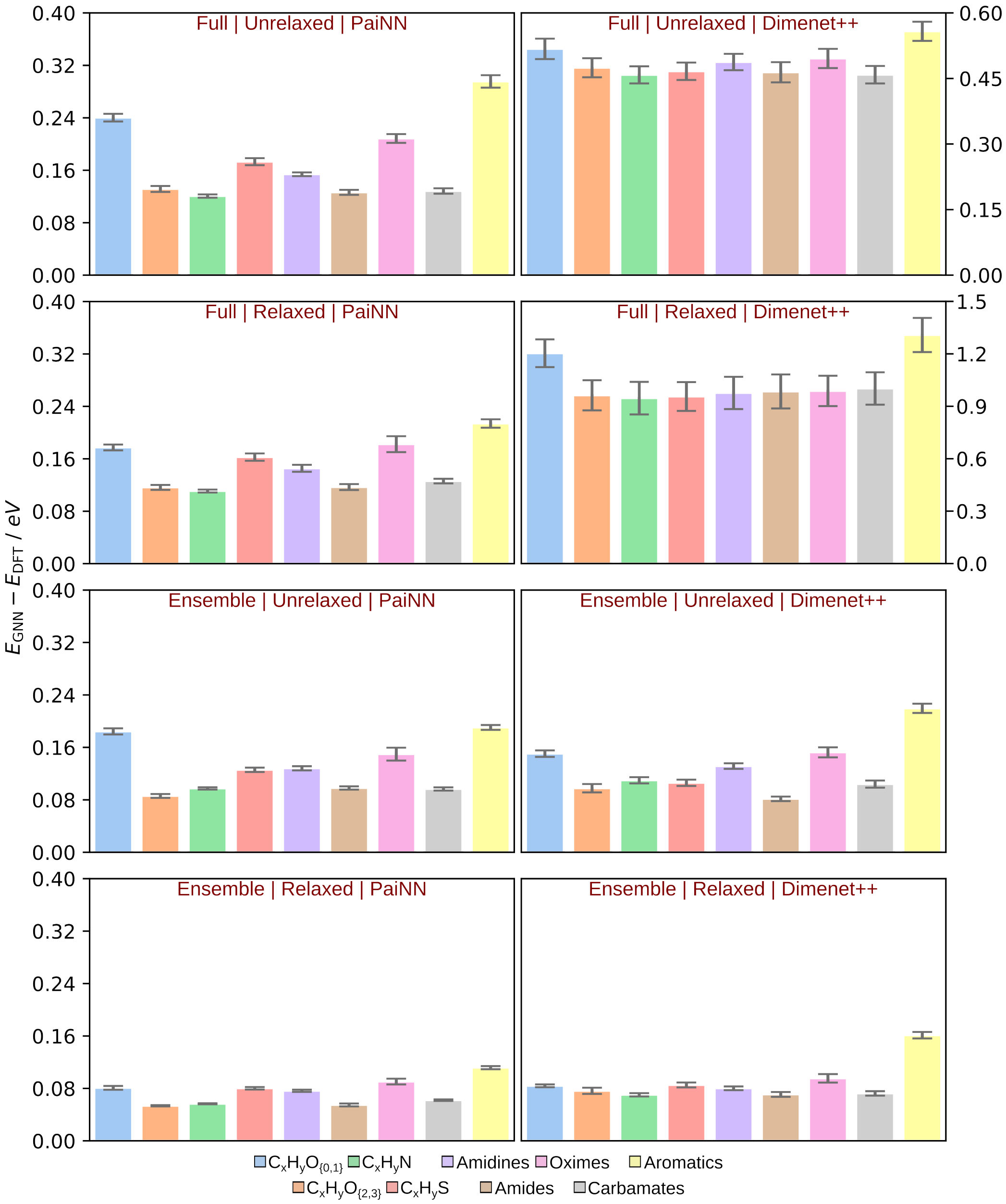

Supplementary Figure 24: GAME-Net benchmark: MAE comparison between PaiNN and DimeNet++. Mean absolute error and standard error of the mean for the predictions obtained by the nested cross validation models grouped by family with PaiNN (left column) and DimeNet++ (right column). Each row represents a setting with specific geometry (unrelaxed/relaxed) and graph representation (full slab/ensemble). The dataset size is n=13028. Data are presented as mean ± standard error.

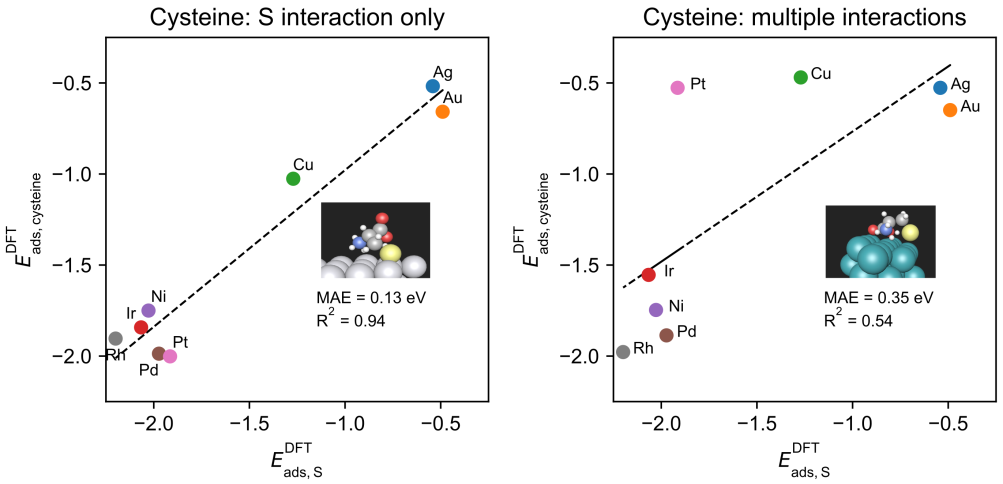

Supplementary Figure 25: Cysteine example study with linear scaling relationships. Left: Scatter-plot of the cysteine DFT adsorption energy as a function of the DFT adsorption energy of S, when the surface interacts only with the S atom. Right: Same scatter-plot, but with the cysteine interacting with the surface through multiple functional groups.

Tables

Supplementary Table 1: FG-dataset: Hydrocarbons, alcohols, aldehydes, ketones and ethers. For each molecule, the IUPAC name and SMILES string are provided.

**IUPAC name**	**SMILES**
Formaldehyde	C=O
Acetylene	C#C
Ethylene	C=C
Acetaldehyde	CC=O
Ethane	CC
Dimethylether	COC
Ethanol	CCO
Propyne	C#CC
Propylene	C=CC
Acetone	CC(=O)C
Propane	CCC
Ethylmethylether	CCOC
N-propanol	CCCO
I-propanol	CC(C)O
2-butyne	CC#CC
1-butyne	C#CCC
1-butene	C=CCC
2-butene-cis	C/C=C\C
2-butene-trans	C/C=C/C
I-butene	CC(=C)C
Butanone	CCC(=O)C
N-butane	CCCC
I-butane	CC(C)C
N-butanol	CCCCO
2-butanol-R	C[C@H](CC)O
2-butanol-S	C[C@@H](CC)O
T-butanol	CC(C)(C)O
Propionaldehyde	CCC=O
Butyraldehyde	CCCC=O
Isobutyraldehyde	CC(C=O)C
Ethoxyethane	CCOCC
1-methoxypropane	CCCOC

Supplementary Table 2: FG-dataset: Carbonates, carboxylic acids and esters. For each molecule, the IUPAC name and SMILES string are provided.

**IUPAC name**	**SMILES**
Formic acid	C(=O)O
Carbonic acid	C(=O)(O)O
Acetic acid	C(=O)(C)O
Methyl formate	C(=O)OC
Methyl hydrogen carbonate	C(=O)(O)OC
Propionic acid	CCC(=O)O
Ethyl formate	C(=O)OCC
Methyl acetate	C(=O)(C)OC
Ethyl hydrogen carbonate	C(=O)(OCC)O
Dimethyl carbonate	C(=O)(OC)OC
Butyric acid	CCCC(=O)O
Isobutyric acid	CC(C(=O)O)C
Propyl formate	C(=O)OCCC
Isopropyl formate	C(=O)OC(C)C
Ethyl acetate	CC(=O)OCC
Methyl propionate	CCC(=O)OC
Propyl hydrogen carbonate	C(=O)(OCCC)O
Isopropyl hydrogen carbonate	C(=O)(O)OC(C)C
Ethyl methyl carbonate	C(=O)(OC)OCC

Supplementary Table 3: FG-dataset: Amines and imines. For each molecule, the IUPAC name and SMILES string are provided.

**IUPAC name**	**SMILES**
Methanimine	C=N
Methanamine	CN
Ethanimine	CC=N
Methylmethanimine	C=NC
Ethanamine	CCN
Dimethylamine	CNC
Propan-1-imine	CCC=N
N-ethylmethanimine	C=NCC
(E)-N-methylethanimine	C/C=N/C
(Z)-N-methylethanimine	C/C=N\C
Propan-2-imine	CC(=N)C
Propan-1-amine	CCCN
Propan-2-amine	CC(C)N
N-methylethanamine	CCNC
Trimethylamine	CN(C)C
Butan-1-imine	CCCC=N
2-methylpropan-1-imine	CC(C)C=N
N-propylmethanimine	C=NCCC
N-isopropylmethanimine	C=NC(C)C
(E)-N-ethylethanimine	C/C=N/CC
(Z)-N-ethylethanimine	C/C=N\CC
(E)-N-methylpropan-1-imine	CC/C=N/C
(Z)-N-methylpropan-1-imine	CC/C=N\C
Butan-2-imine	CCC(=N)C
N-methylpropan-2-imine	CC(=NC)C
Butan-1-amine	CCCCN
(R)-butan-2-amine	C[C@H](CC)N
(S)-butan-2-amine	C[C@@H](CC)N
2-methylpropan-2-amine	CC(C)(C)N
Diethylamine	CCNCC
N-methylpropan-1-amine	CCCNC
N,N-dimethylethanamine	CCN(C)C

Supplementary Table 4: FG-dataset: Amidines. For each molecule, the IUPAC name and SMILES string are provided.

**IUPAC name**	**SMILES**
Formimidamide	C(=N)N
N-methylformimidamide	C(=N)NC
acetimidamide	C(=N)(C)N
(E)-N'-methylformimidamide	C(=[NH2])[N]C
(Z)-N'-methylformimidamide	C(=[NH2])[N]C
N-ethylformimidamide	C(=N)NCC
Propionimidamide	C(=N)(CC)N
(E)-N'-ethylformimidamide	C(=[NH2])[N]CC
(Z)-N'-ethylformimidamide	C(=[NH2])[N]CC
(E)-N'-methylacetimidamide	C(=[NH2])(C)[N]C
(Z)-N'-methylacetimidamide	C(=[NH2])(C)[N]C
N,N-dimethylformimidamide	C(=N)N(C)C
N-methylacetimidamide	C(=N)(C)NC
(E)-N,N'-dimethylformimidamide	C(=N\C)/NC
(Z)-N,N'-dimethylformimidamide	C(=N\C)\NC
N-isopropylformimidamide	C(=N)NC(C)C
N-propylformimidamide	C(=N)NCCC
Isobutyrimidamide	C(=N)(C(C)C)N
Butyrimidamide	C(=N)(CCC)N
(E)-N'-isopropylformimidamide	C(=[NH2])[N]C(C)C
(Z)-N'-isopropylformimidamide	C(=[NH2])[N]C(C)C
(E)-N'-propylformimidamide	C(=[NH2])[N]CCC
(Z)-N'-propylformimidamide	C(=[NH2])[N]CCC
(E)-N'-ethylacetimidamide	C(=[NH2])(C)[N]CC
(Z)-N'-ethylacetimidamide	C(=[NH2])(C)[N]CC
(E)-N'-methylpropionimidamide	C(=[NH2])(CC)[N]C
(Z)-N'-methylpropionimidamide	C(=[NH2])(CC)[N]C
N-ethyl-N-methylformimidamide	C(=N)N(C)CC
N-methylpropionimidamide	C(=N)(CC)NC
N-ethylacetimidamide	C(=N)(C)NCC
N,N-dimethylacetimidamide	C(=N)(C)N(C)C
(E)-N'-ethyl-N-methylformimidamide	C(=N\CC)/NC
(Z)-N'-ethyl-N-methylformimidamide	C(=N\CC)\NC
(E)-N-ethyl-N'-methylformimidamide	C(=N\C)/NCC
(Z)-N-ethyl-N'-methylformimidamide	C(=N\C)\NCC
(E)-N,N,N'-trimethylformimidamide	C(=N\C)/N(C)C
(Z)-N,N,N'-trimethylformimidamide	C(=N\C)\N(C)C
(E)-N,N'-dimethylacetimidamide	C(=N\C)(\C)/NC
(Z)-N,N'-dimethylacetimidamide	C(=N\C)(/C)\NC

Supplementary Table 5: FG-dataset: Thiols, thials, thioketones and thioethers. For each molecule, the IUPAC name and SMILES string are provided.

**IUPAC name**	**SMILES**
Methanethial	C=S
Methanethiol	CS
Ethanethial	CC=S
Ethanethiol	CCS
Dimethylsulfane	CSC
Propanethial	CCC=S
Propane-2-thione	CC(=S)C
Propane-1-thiol	CCCS
Propane-2-thiol	CC(C)S
Ethyl(methyl)sulfane	CCSC
Butanethial	CCCC=S
2-methylpropanethial	CC(C)C=S
Butane-2-thione	CCC(=S)C
Butane-1-thiol	CCCCS
(R)-butane-2-thiol	C[C@H](CC)S
(S)-butane-2-thiol	C[C@@H](CC)S
2-methylpropane-2-thiol	CC(C)(C)S
Diethylsulfane	CCSCC
Methyl(propyl)sulfane	CCCSC

Supplementary Table 6: FG-dataset: Amides. For each molecule, the IUPAC name and SMILES string are provided.

IUPAC name	SMILES
Formamide	C(=O)N
N-methylformamide	C(=O)NC
Acetamide	C(=O)(C)N
N-ethylformamide	C(=O)NCC
Propionamide	C(=O)(CC)N
N,N-dimethylformamide	C(=O)N(C)C
N-methylacetamide	C(=O)(C)NC
N-isopropylformamide	C(=O)NC(C)C
N-propylformamide	C(=O)NCCC
Isobutyramide	C(=O)(C(C)C)N
Butyramide	C(=O)(CCC)N
N-ethyl-N-methylformamide	C(=O)N(C)CC
N-ethylacetamide	C(=O)(C)NCC
N-methylpropionamide	C(=O)(CC)NC
N,N-dimethylacetamide	C(=O)(C)N(C)C

Supplementary Table 7: FG-dataset: Oximes. For each molecule, the IUPAC name and SMILES string are provided.

IUPAC name	SMILES
Formaldehyde oxime	C=NO
(E)-acetaldehyde oxime	C(=N\O)/C
(Z)-acetaldehyde oxime	C(=N\O)\C
(E)-propionaldehyde oxime	C(=N\O)/CC
(Z)-propionaldehyde oxime	C(=N\O)\CC
Propan-2-one oxime	C(=NO)(C)C
(E)-butyraldehyde oxime	C(=N\O)/CCC
(Z)-butyraldehyde oxime	C(=N\O)\CCC
(E)-isobutyraldehyde oxime	C(=N\O)/C(C)C
(Z)-isobutyraldehyde oxime	C(=N\O)\C(C)C
(E)-butan-2-one oxime	C(=N\O)(\C)/CC
(Z)-butan-2-one oxime	C(=N\O)(\CC)/C

Supplementary Table 8: FG-dataset: Carbamate esters. For each molecule, the IUPAC name and SMILES string are provided.

**IUPAC name**	**SMILES**
carbamic acid	C(=O)(N)O
methylcarbamic acid	C(=O)(NC)O
methyl carbamate	C(=O)(N)OC
ethylcarbamic acid	C(=O)(NCC)O
ethyl carbamate	C(=O)(N)OCC
dimethylcarbamic acid	C(=O)(N(C)C)O
methyl methylcarbamate	C(=O)(NC)OC
isopropylcarbamic acid	C(=O)(NC(C)C)O
propylcarbamic acid	C(=O)(NCCC)O
isopropyl carbamate	C(=O)(N)OC(C)C
propyl carbamate	C(=O)(N)OCCC
ethyl(methyl)carbamic acid	C(=O)(N(CC)C)O
methyl ethylcarbamate	C(=O)(NCC)OC
ethyl methylcarbamate	C(=O)(NC)OCC
methyl dimethylcarbamate	C(=O)(N(C)C)OC

Supplementary Table 9: FG-dataset: Aromatic molecules. For each molecule, the IUPAC name and SMILES string are provided.

**IUPAC name**	**SMILES**
Furan	o1cccc1
Thiophene	s1cccc1
Pyrrole	[nH]1cccc1
Pyridine	c1ccncc1
Cyclopentadiene	C1C=CC=C1
Benzene	c1ccccc1
Phenol	Oc1ccccc1
Thiophenol	Sc1ccccc1
Aniline	Nc1ccccc1
Toluene	Cc1ccccc1
Para-xylene	Cc1ccc(C)cc1
Meta-xylene	Cc1cccc(C)c1
Ortho-xylene	Cc1ccccc1C
Benzofuran	o1ccc2ccccc12
Isobenzofuran	o1cc2ccccc2c1
Benzo[b]thiophene	s1ccc2ccccc12
Benzo[c]thiophene	s1cc2ccccc2c1
1H-indole	[nH]1ccc2ccccc12
2H-indole	C1C=C2C=CC=CC2=N1
Quinoline	c1ccc2ncccc2c1
Isoquinoline	c1ccc2cnccc2c1
1H-indene	C1C=Cc2ccccc12
2H-indene	C1C=C2C=CC=CC2=C1
Naphthalene	c1ccc2ccccc2c1

Supplementary Table 10: Hyperparameter optimization summary. For each studied hyperparameter, the type, search range and optimum value are listed. All the pooling-wise options refer to the GMT implementation in Pytorch Geometric 2.0.3 (PyG).

**Hyperparameter**	**Type**	**Search space**	**Optimum**
Batch size	int	16, 32, 64, 128	16
Loss function	func	MAE, MSE	MAE
Initial lr	float	1e-1, 1e-2, 1e-3, 1e-4	1e-3
Lr-patience	int	5, 7, 9	5
Lr-factor	float	0.5, 0.7, 0.9	0.7
Minimum lr	float	1e-7, 1e-8, 1e-9	1e-8
Amsgrad	bool	True, False	True
Layers width	int	64:8:256	160
Bias inclusion	bool	True, False	False
Linear layers	int	0, 1, 2, 3, 4	1
Convolutional layers	int	1, 2, 3, 4, 5	3
Convolution type	func	GraphSAGE, GATv2	GraphSAGE
Normalized conv.	bool	True, False	False
Root-weighted conv.	bool	True, False	True
Pool ratio	float	0.25, 0.50, 0.75	0.25
Pool heads	int	1, 2, 4	1
Pool sequence	list[func]	(see PyG Docs)	["GMPool_I"]
Pool normalization	bool	True, False	False

Supplementary Table 11: FG-dataset: Cross validation MAE and SEM *per* family. Mean and standard error of the mean of the absolute error of the models obtained by nested cross validation sorted by chemical family. Values are reported in eV.

**Chemical Family**	**Mean**	**Standard Error**
$C_xH_yO_{(0,1)}$	0.20	0.0123
$C_xH_yO_{(2,3)}$	0.12	0.0185
C_xH_yN	0.14	0.0107
C_xH_yS	0.19	0.0177
Amidines	0.15	0.0065
Amides	0.11	0.0061
Oximes	0.18	0.0150
Carbamates	0.13	0.0070
Aromatics	0.34	0.0162

Supplementary Table 12: FG-dataset: Cross validation MAE and SEM errors *per* metal. Mean and standard error of the mean of the absolute error of the models obtained by nested cross validation sorted by metal (the last row considers the gas-phase subset). Values are reported in eV.

**Metal**	**Mean**	**Standard Error**
Ag	0.15	0.0125
Au	0.17	0.0102
Cd	0.13	0.0163
Co	0.20	0.0083
Cu	0.13	0.0096
Fe	0.20	0.0109
Ir	0.19	0.0170
Ni	0.17	0.0185
Os	0.18	0.0075
Pd	0.14	0.0086
Pt	0.19	0.0179
Rh	0.16	0.0110
Ru	0.18	0.0138
Zn	0.11	0.0120
Gas	0.39	0.0892

Supplementary Table 13: GAME-Net predictions on the BM-dataset. Adsorption energy of the BM-dataset samples obtained with DFT and GAME-Net. Values are reported in eV.

**Molecule**	**Family**	**Metal**	$\mathbf{E_{ads}^{DFT}}$	$\mathbf{E_{ads}^{GNN}}$	**Absolute error**
mol1	Polyurethanes	Ag	-0.75	-0.06	0.69
mol2	Polyurethanes	Ag	-0.81	-0.25	0.56
mol3	Polyurethanes	Ag	-0.77	-0.23	0.54
mol4	Polyurethanes	Ag	-0.84	-0.37	0.47
mol5	Polyurethanes	Ag	-1.11	-0.66	0.45
mol1	Polyurethanes	Au	-0.50	0.00	0.50
mol2	Polyurethanes	Au	-0.40	-0.34	0.06
mol3	Polyurethanes	Au	-0.36	-0.12	0.24
mol4	Polyurethanes	Au	-0.39	0.00	0.39
mol5	Polyurethanes	Au	-0.84	-0.41	0.43
PE	Plastics	Pt	-1.17	-0.95	0.22
PP-it	Plastics	Pt	-0.93	-0.40	0.53
PP-st	Plastics	Pt	-0.86	-0.15	0.71
PS	Plastics	Pt	-2.95	-2.81	0.14
PET	Plastics	Pt	-2.86	-2.13	0.73
PE	Plastics	Ru	-0.32	-0.47	0.15
PP-it	Plastics	Ru	-0.19	-0.33	0.14
PP-st	Plastics	Ru	0.01	-0.26	0.27
PS	Plastics	Ru	-3.10	-3.92	0.82
PET	Plastics	Ru	-4.78	-4.55	0.23
mol1	Biomass	Ni	-2.86	-3.32	0.46
mol2	Biomass	Ni	-2.39	-3.05	0.66
mol3	Biomass	Ni	-2.79	-1.85	0.94
mol4	Biomass	Ni	-1.92	-1.24	0.68
mol5	Biomass	Ni	-1.60	-1.04	0.56
mol1	Biomass	Ru	-3.65	-4.19	0.54
mol2	Biomass	Ru	-3.15	-3.78	0.63
mol3	Biomass	Ru	-3.41	-2.58	0.83
mol4	Biomass	Ru	-2.50	-2.10	0.40
mol5	Biomass	Ru	-1.99	-1.40	0.59

Supplementary Table 14: GAME-Net benchmark on literature datasets. The MAE and RMSE for the adsorption energy are reported in eV.

**Dataset**	**N**	**Adsorbates**	**MAE**	**RMSE**
Andersen et al. [6]	506	open-shell	1.49	1.93
García-Muelas et al. [7]	924	open-shell	0.85	1.17
Pablo-García et al. [8]	879	open-shell	0.58	0.80
**FG-dataset**	3108	closed-shell	0.18	0.32
**BM-dataset**	30	closed-shell	0.48	0.53
**fcc(110)**	1776	closed-shell	0.42	0.56
**fcc(100)**	1776	closed-shell	0.34	0.51

Supplementary Table 15: GAME-Net benchmark against PaiNN, DimeNet++ and GemNet-dT. "(*)" in the model name refers to the original model implementation. All values refer to the prediction of the adsorption energy. When "neighbours", the graph representation includes only the metal atoms directly interacting with the adsorbate. Values for MAE and SEM are in eV.

			**FG-dataset**		**BM-dataset**	
**Model**	**Graph**	**Geometry**	**MAE**	**SEM**	**MAE**	**R^2**
PaiNN(*)	full slab	unrelaxed	0.18	1.86E-3	2.86	< 0
DimeNet++(*)	full slab	unrelaxed	0.49	1.62E-2	11.91	< 0
PaiNN	full slab	relaxed	0.15	2.16E-3	4.33	< 0
DimeNet++	full slab	relaxed	1.06	8.36E-2	14.00	< 0
PaiNN	neighbours	unrelaxed	0.13	1.31E-3	0.44	0.54
DimeNet++	neighbours	unrelaxed	0.14	2.53E-3	0.46	0.65
PaiNN	neighbours	relaxed	0.08	3.80E-4	0.44	0.81
DimeNet++	neighbours	relaxed	0.09	1.86E-3	0.46	0.81
GemNet-dT	full slab	unrelaxed	0.13	2.26E-3	3.84	< 0
GemNet-dT	neighbours	unrelaxed	0.13	1.63E-3	0.95	0.69
**GAME-Net**	neighbours	relaxed	0.18	2.00E-3	0.48	0.82

Supplementary Table 16: Technical comparison between GAME-Net and benchmark models.

Model	Trainable Parameters	Training Time	Training Hardware
PaiNN	72,255,495	$\approx$ 3600 s	NVIDIA A100-SXM 80GB
DimeNet++	2,755,462	$\approx$ 2000 s	NVIDIA A100-SXM 80GB
GemNet-dT	23,176,469	$\approx$ 16000 s	NVIDIA A100-SXM 80GB
GAME-Net	285,761	$\approx$ 300 s	NVIDIA GeForce MX450 2GB

Supplementary Table 17: Comparison of GAME-Net predictions with experimental adsorption energies (ZPE-corrected) of two example systems from Campbell & co.[14] Values are in eV.

Molecule	Surface	$\mathbf{E_{ads}^{exp}}$	$\mathbf{E_{ads}^{GNN}}$
C_3H_8	Pt(111)	-0.39	-0.41
n-C_4H_{10}	Pt(111)	-0.48	-0.61

References

1. García-Muelas, R. & López, N. Collective Descriptors for the Adsorption of Sugar Alcohols on Pt and Pd(111). *The Journal of Physical Chemistry C* **118,** 17531–17537. doi:10.1021/jp502819s (2014).

2. García-Muelas, R., Li, Q. & López, N. Density Functional Theory Comparison of Methanol Decomposition and Reverse Reactions on Metal Surfaces. *ACS Catalysis* **5,** 1027–1036. doi:10.1021/cs501698w. http://dx.doi.org/10.1021/cs501698w (2015).

3. Li, Q. & López, N. Chirality, Rigidity, and Conjugation: a First-Principles Study of the Key Molecular Aspects of Lignin Depolymerization on Ni-Based Catalysts. *ACS Catalysis* **8,** 4230–4240. doi:10.1021/acscatal.8b00067. http://dx.doi.org/10.1021/acscatal.8b00067 (2018).

4. Puértolas, B., Rellán-Piñeiro, M., Núñez-Rico, J. L., Amrute, A. P., Vidal-Ferran, A., López, N., Pérez-Ramírez, J. & Wershofen, S. Mechanistic Insights Into the Ceria-Catalyzed Synthesis of Carbamates As Polyurethane Precursors. *ACS Catalysis* **9,** 7708–7720. doi:10.1021/acscatal.9b02086. http://dx.doi.org/10.1021/acscatal.9b02086 (2019).

5. Ding, S., Hülsey, M. J., Pérez-Ramírez, J. & Yan, N. Transforming Energy With Single-Atom Catalysts. *Joule* **3,** 2897–2929. doi:10.1016/j.joule.2019.09.015. http://dx.doi.org/10.1016/j.joule.2019.09.015 (2019).

6. Andersen, M., Levchenko, S. V., Scheffler, M. & Reuter, K. Beyond Scaling Relations for the Description of Catalytic Materials. *ACS Catalysis* **9,** 2752–2759. doi:10.1021/acscatal.8b04478. eprint: https://doi.org/10.1021/acscatal.8b04478. https://doi.org/10.1021/acscatal.8b04478 (2019).

7. García-Muelas, R. & López, N. Statistical learning goes beyond the d-band model providing the thermochemistry of adsorbates on transition metals. *Nature Communications* **10,** 4687. ISSN: 2041-1723. doi:10.1038/s41467-019-12709-1. https://doi.org/10.1038/s41467-019-12709-1 (2019).

8. Pablo-García, S., Veenstra, F. L. P., Ting, L. R. L., García-Muelas, R., Dattila, F., Martín, A. J., Yeo, B. S., Pérez-Ramírez, J. & López, N. Mechanistic Routes Toward C_3 Products in Copper-Catalysed CO_2 Electroreduction. *Catalysis Science & Technology* **12,** 409–417. doi:10.1039/d1cy01423d. http://dx.doi.org/10.1039/D1CY01423D (2022).

9. Schütt, K., Unke, O. & Gastegger, M. *Equivariant message passing for the prediction of tensorial properties and molecular spectra* in *Proceedings of the 38th International Conference on Machine Learning* (eds Meila, M. & Zhang, T.) **139** (PMLR, 18–24 Jul 2021), 9377–9388. https://proceedings.mlr.press/v139/schutt21a.html.

10. Gasteiger, J., Groß, J. & Günnemann, S. Directional Message Passing for Molecular Graphs. *CoRR.* doi:10.48550/arXiv.2003.03123. arXiv: 2003.03123. http://arxiv.org/abs/2003.03123v2 (2020).

11. Gasteiger, J., Becker, F. & Günnemann, S. *GemNet: Universal Directional Graph Neural Networks for Molecules* in *Advances in Neural Information Processing Systems* (eds Ranzato, M., Beygelzimer, A., Dauphin, Y., Liang, P. & Vaughan, J. W.) **34** (Curran Associates, Inc., 2021), 6790–6802. doi:10.48550/arXiv.2106.08903.

12. Tran, R., Lan, J., Shuaibi, M., Goyal, S., Wood, B. M., Das, A., Heras-Domingo, J., Kolluru, A., Rizvi, A., Shoghi, N., Sriram, A., Ulissi, Z. & Zitnick, C. L. The Open Catalyst 2022 (OC22) Dataset and Challenges for Oxide Electrocatalysis. *CoRR.* doi:10.48550/arXiv.2206.08917. arXiv: 2206.08917 [cond-mat.mtrl-sci]. http://arxiv.org/abs/2206.08917v1 (2022).

13. *Open Catalyst Project code repository in Github. [00c4c6b]* https://github.com/Open-Catalyst-Project/ocp.

14. Wellendorff, J., Silbaugh, T. L., Garcia-Pintos, D., Nørskov, J. K., Bligaard, T., Studt, F. & Campbell, C. T. A benchmark database for adsorption bond energies to transition metal surfaces and comparison to selected DFT functionals. *Surface Science* **640,** 36–44. ISSN: 0039-6028. doi:https://doi.org/10.1016/j.susc.2015.03.023. https://www.sciencedirect.com/science/article/pii/S0039602815000837 (2015).
